# Human Bone Marrow-Resident Natural Killer Cells Have a Unique Transcriptional Profile and Resemble Resident Memory CD8^+^ T Cells

**DOI:** 10.3389/fimmu.2018.01829

**Published:** 2018-08-22

**Authors:** Janine E. Melsen, Gertjan Lugthart, Carly Vervat, Szymon M. Kielbasa, Sander A. J. van der Zeeuw, Henk P. J. Buermans, Monique M. van Ostaijen-ten Dam, Arjan C. Lankester, Marco W. Schilham

**Affiliations:** ^1^Department of Pediatrics, Leiden University Medical Center, Leiden, Netherlands; ^2^Department of Biomedical Data Sciences, Leiden University Medical Center, Leiden, Netherlands; ^3^Department of Human Genetics, Leiden Genome Technology Center, Leiden University Medical Center, Leiden, Netherlands

**Keywords:** natural killer cells, tissue-resident, RNA sequence, CD8^+^ T cells, lymphoid tissue

## Abstract

Human lymphoid tissues harbor, in addition to CD56^bright^ and CD56^dim^ natural killer (NK) cells, a third NK cell population: CD69^+^CXCR6^+^ lymphoid tissue (lt)NK cells. The function and development of ltNK cells remain poorly understood. In this study, we performed RNA sequencing on the three NK cell populations derived from bone marrow (BM) and blood. In ltNK cells, 1,353 genes were differentially expressed compared to circulating NK cells. Several molecules involved in migration were downregulated in ltNK cells: *S1PR1, SELPLG* and *CD62L*. By flow cytometry we confirmed that the expression profile of adhesion molecules (CD49e^−^, CD29^low^, CD81^high^, CD62L^−^, CD11c^−^) and transcription factors (Eomes^high^, Tbet^low^) of ltNK cells differed from their circulating counterparts. LtNK cells were characterized by enhanced expression of inhibitory receptors TIGIT and CD96 and low expression of DNAM1 and cytolytic molecules (*GZMB, GZMH, GNLY*). Their proliferative capacity was reduced compared to the circulating NK cells. By performing gene set enrichment analysis, we identified DUSP6 and EGR2 as potential regulators of the ltNK cell transcriptome. Remarkably, comparison of the ltNK cell transcriptome to the published human spleen-resident memory CD8^+^ T (Trm) cell transcriptome revealed an overlapping gene signature. Moreover, the phenotypic profile of ltNK cells resembled that of CD8^+^ Trm cells in BM. Together, we provide transcriptional and phenotypic data that clearly distinguish ltNK cells from both the CD56^bright^ and CD56^dim^ NK cells and substantiate the view that ltNK cells are tissue-resident cells, which are functionally restrained in killing and have low proliferative activity.

## Introduction

The recent identification of tissue-resident lymphocytes in both human and mice contributes to our extending knowledge on the heterogeneity of innate and adaptive lymphocyte populations. One of the key markers expressed by the majority of these non-circulating lymphocytes is CD69, previously known as an early activation marker. Nowadays, it has become clear that CD69 is associated with tissue-residency because it promotes internalization of sphingosine-1-phosphate receptor 1 (S1PR1) (1–3). Expression of S1PR1 enables migration of lymphocytes towards the S1P gradient, which is higher in blood compared to tissues (4, 5).

Subsets of the human innate natural killer (NK) cells exhibit a tissue-resident phenotype as demonstrated in various organs, including the uterus, liver, tonsils, bone marrow (BM), spleen, and lymph nodes (6–12). CD69^+^ tissue-resident NK cells are characterized by a distinct chemokine receptor and adhesion molecule repertoire (13, 14). For instance, CXCR6 is highly expressed by tissue-resident NK cells in lymphoid tissues and liver, while CD49a is characteristic for uterus-resident NK cells (6, 7, 9, 12). The remaining non-resident (circulating) NK cells are subdivided into two populations based on CD56 and CD16 expression: CD56^bright^CD16^−/+^ and CD56^dim^CD16^+^ (15). The CD56^bright^CD16^−/+^ NK cells are potent cytokine producers (IFN-γ, TNF-α), while the CD56^dim^CD16^+^ NK cells have a high capacity to kill infected and transformed cells (16–18). The function of tissue-resident NK cells is still unknown, since they are neither good producers of IFN-γ nor potent killers (7, 12).

Among the human tissue-resident lymphocytes of the adaptive immune system, the tissue-resident memory (Trm) cells are extensively investigated and have been identified in skin, spleen, tonsil, lung, liver, lymph node, salivary glands, and intestines (19–25). Trm cells exhibit a unique molecular program, which distinguishes them from their circulating counterparts (22, 23, 25–29). Local environmental cues appear to play a role in the development of Trm; numerous murine studies provided evidence for a developmental pathway in which KLRG1^−^ CD8^+^ effector T cells give rise to Trm cells under influence of local cues, such as TGF-β and IL-15 (27, 30–32). In contrast to the Trm cells, transcriptional regulation and developmental requirements for tissue-resident NK cells are poorly understood.

In this study, we demonstrated that lymphoid tissue-resident (lt)NK cells in BM possess a transcriptional profile, which distinguished them from the two circulating NK cell subsets. In addition, by the use of published data, we found that lymphoid tissue-resident CD69^+^CD8^+^ Trm cells share a transcriptional and phenotypic profile with ltNK cells. Together, we provide a comprehensive molecular framework of the conventional CD56^bright^ and CD56^dim^ NK cells as well as the tissue-resident ltNK cells and provide a core gene signature, which might be involved in promoting tissue-residency.

## Materials and Methods

### Tissue and Ethics Statement

With approval of the institutional review board (P08.001), residual paired BM and blood samples from four healthy BM donors were used for RNA sequencing after informed consent was provided. Validation of the RNA sequence data by flow cytometry was performed by analyzing residual BM samples of healthy donors (*n* = 18) and was evaluated anonymously in accordance with Dutch national ethical and professional guidelines (http://www.federa.org).

### Purification of NK Cell Populations

**Figure 1:**
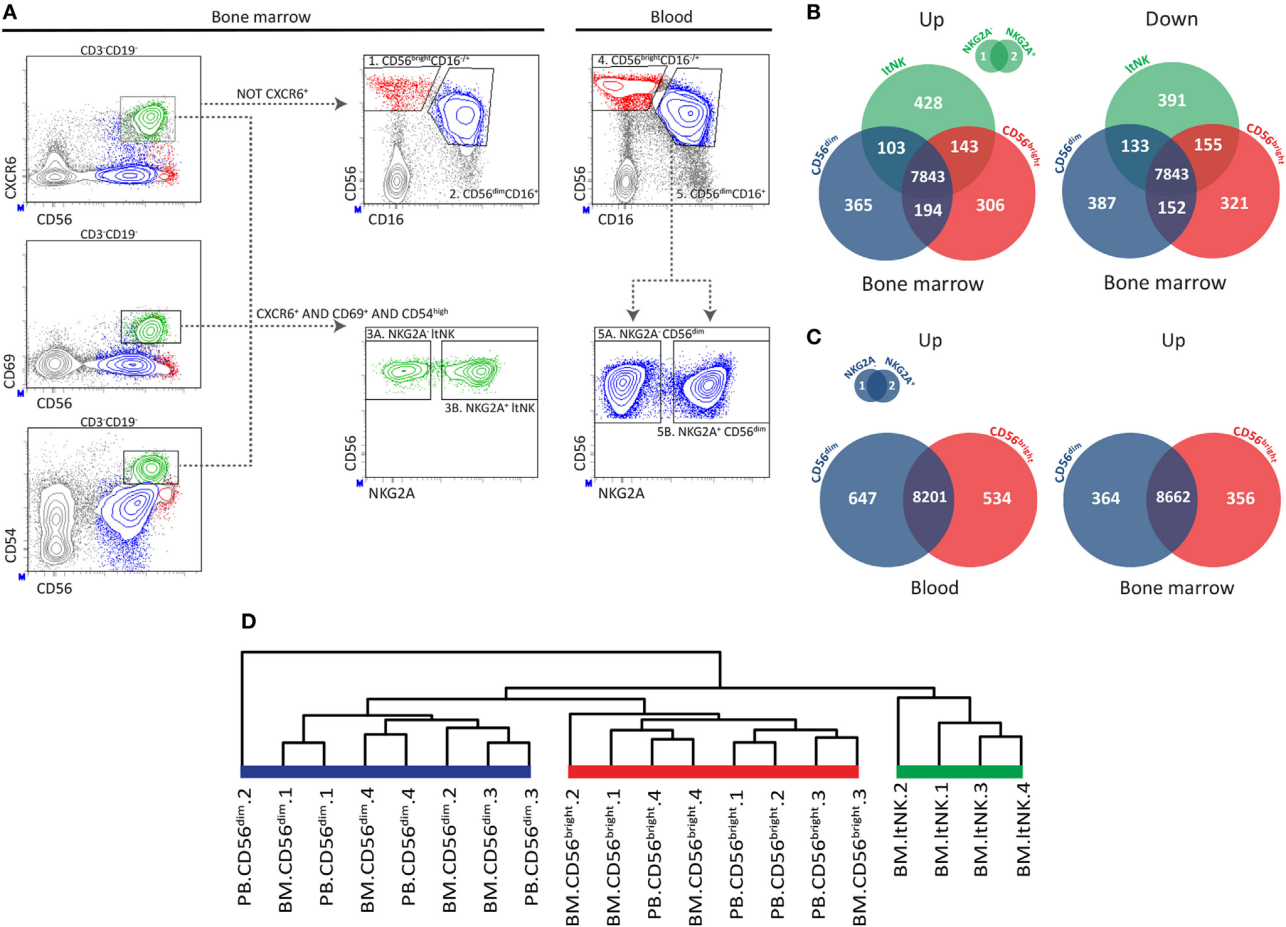
RNA sequence analysis confirms distinctiveness of CD56^bright^, CD56^dim^, and ltNK cells. **(A)** The applied gating strategy to isolate the different natural killer (NK) cell subsets from bone marrow (BM) and blood is depicted. A representative sample of BM NK cell enriched mononuclear cells is shown. Lymphocytes were gated based on forward and sideward scatter and the doublets were excluded. Residual B and T cells were excluded by selecting CD3^−^CD19^−^ lymphocytes. LtNK cells were defined as CD56^+^CXCR6^+^CD69^+^CD54^+^. CD56^+^CXCR6^−^ NK cells were divided into CD56^bright^CD16^+/−^ and CD56^dim^CD16^+^ NK cells. The blood-derived CD56^dim^ and BM derived ltNK cells were further divided into a NKG2A^+^ and NKG2A^−^ fraction. **(B,C)** Venn-diagrams illustrating the number of differentially expressed genes (up- and downregulated) between the NK cell subsets in blood and BM. **(D)** Unsupervised hierarchical clustering of the individual donor samples of BM and blood (PB) derived CD56^bright^, CD56^dim^, and ltNK cells. The clustering is based on the expression pattern of all the 9,382 genes, which were included in the differential expression analysis.

Mononuclear cells (MNCs) were isolated by Ficoll-Isopaque (Leiden University Medical Center Pharmacy, Leiden, Netherlands) density gradient centrifugation, and NK cells were enriched using the MACS untouched NK cell isolation kit (Miltenyi Biotec, Bergisch Gladbach, Germany) according to manufacturers’ instructions. Lymphocytes were stained for surface markers with the antibodies listed in Table S1 in Supplementary Material. The detailed staining procedure is provided in the supplemental methods. The following NK cell populations were isolated from the BM: NKG2A^+^ ltNK cells, NKG2A^−^ ltNK cells, CD56^bright^ and CD56^dim^ NK cells. From blood, the CD56^bright^, NKG2A^+^CD56^dim^, and NKG2A^−^CD56^dim^ NK cells were isolated. The populations were FACS-purified on an ARIAIII cell sorter [Becton Dickinson (BD), Franklin Lakes, NJ, USA]. The gating strategy is depicted in Figure 1A. The NK cell populations were collected in NucleoSpin RA1 lysis buffer (Machery Nagel, Düren, Germany) and stored at −80°C prior to analyses. The total numbers of cells analyzed for each purified NK cell population are provided in Table S2 in Supplementary Material.

### RNA Sequence

Total RNA was extracted using the NucleoSpin RNA XS kit (Macherey Nagel) and converted to cDNA and pre-amplified using the SMARTer Ultra Low RNA kit (Clontech Laboratories, Mountain View, CA, USA). A sequencing library was generated from 1 ng amplified cDNA using the Illumina Nextera XT kit (Illumina, San Diego, CA, USA). All 28 samples were pooled and sequenced on two lanes of the HiSeq2500 system (Illumina). A detailed description of the methods used to perform RNA sequencing and map the reads are provided in the supplemental methods. RNA paired-end reads were aligned to the *Homo Sapiens* reference genome version hg19 using GSNAP. The read counts were normalized for library size with the “voom” function of the limma package (33). Genes with averaged normalized counts below 4 counts per million of uniquely mapped reads (CPM) were excluded from further analysis. To determine the differentially expressed genes, a linear model was fitted to each gene and empirical Bayes moderated *t*-statistics were applied. Genes were considered as significantly differentially expressed if the false discovery rate (FDR) was below the 0.05 threshold. Unsupervised hierarchical clustering was performed using the hclust function in R (Version 0.99.903, R Foundation for Statistical Computing, Vienna, Austria). Heatmaps were generated using the heatmap3 package (34, 35).

### RNA Sequence Validation by Flow Cytometry

To validate the differentially expressed genes at the protein level, MNCs isolated from blood and BM were stained with fluorochrome-labeled antibodies in PBS (Braun, Melsungen, Germany) containing 0.1% NaN_3_ (LUMC Pharmacy) and 0.5% bovine serum albumin (BSA, Sigma-Aldrich, St. Louis, MO, USA). When unconjugated anti-CXCR6 was used, a three-step staining procedure was used as described under the Section “Purification of NK Cell Populations” in supplemental methods. To determine intracellular expression of Eomes and Tbet, MNCs were fixated and permeabilized using the FOXP3 transcription factor staining kit, as described in the protocol (Invitrogen, Carlsbad, CA, USA). The antibodies used are listed in Table S3 in Supplementary Material. Data were acquired on a LSRII flow cytometer (BD) using FACS Diva software (v8.0, BD). In the extracellular and intracellular staining protocols, respectively DAPI (25 ng/ml, Sigma-Aldrich) and Fixable Viability Dye eFluor 455UV (1:1,000, eBioscience, San Diego, CA, USA), were used to exclude dead cells. Kaluza analysis software (v1.5, Beckman Coulter, Brea, CA, USA) was used for post-acquisition analysis. Density plots were generated using FACS Diva software (v8.0). NK cells were defined as living CD45^+^CD19^−^CD3^−^CD7^+^CD56^+^ lymphocytes. Within this cell population, ltNK cells were identified as CD69^+^CXCR6^+^ or CD69^+^CD54^high^. The remaining NK cells were divided into CD56^bright^CD16^−/+^ and CD56^dim^CD16^+^ NK cells, based on the levels of CD56 and CD16 expression. CD8^+^ memory T cells were defined as CD3^+^CD8^+^CCR7^−^CD45RA^+/−^ lymphocytes within the live gate (Figure S1 in Supplementary Material). CD69 was used to identify CD8^+^ tissue-resident memory T cells. Fluorescence minus one controls were used as a negative control.

### *In Vitro* Assays

To determine, LIGHT, CD30L, and IFN-γ expression, MNCs from BM were cultured in AIM-V (Thermo Fisher Scientific, Waltham, MA, USA) containing 10% human serum and stimulated with recombinant human IL12 (10 ng/ml, PeproTech, Rocky Hill, NJ, USA), recombinant human IL15 (10 ng/ml, CellGenix, Freiburg, Germany), and recombinant human IL18 (20 ng/ml, MBL International, Woburn, MA, USA), or a combination of phorbol myristate acetate (PMA, 12.5 ng/ml, Sigma-Aldrich), and ionomycin (1 μg/ml, Sigma-Aldrich). BD Golgistop (1:1,500, BD) was added after 1 h of culture. After 4 h of stimulation, cells were harvested and stained for surface markers (Table S3 in Supplementary Material). To stain intracellular IFN-γ, cells were subsequently fixated with 4% paraformaldehyde and permeabilized with saponin, as previously described (Table S3 in Supplementary Material) (36). To study the proliferative capacity ltNK (CD49e^−^CD56^+^CD69^+^CXCR6^+^), CD56^bright^ (CD49e^+^CD56^bright^) and CD56^dim^ (CD49e^+^CD56^dim^CD16^+^) NK cells were purified and cultured for 6 days in the presence of IL2 (1,000 IU/ml, Chiron, Emryville, CA, USA), IL15 (10 ng/ml), or IL21 (10 ng/ml, PeproTech). After 6 days, intracellular Ki67 expression was determined. For this purpose, NK cells were fixated and permeabilized using the FOXP3 transcription factor staining kit (Table S3 in Supplementary Material). The counts of CD56^+^ NK cells after culture were assessed by flow cytometry.

### Gene Set Enrichment Analysis

To determine whether certain gene sets were enriched in the ltNK cell population, CAMERA (limma package) analysis was applied using the normalized expression values of 9,382 genes (37). Gene set collections C2 (curated gene sets), C3 (motif gene sets), C5 (GO gene sets), and C7 (immunologic signatures), derived from the Molecular Signatures Database (MSigDB v6.0) were included. Two analyses were performed: ltNK versus CD56^bright^ and ltNK versus CD56^dim^. Gene sets that were significantly enriched (FDR < 0.05) in both analyses are described in Table S4A in Supplementary Material. The combined *Z*-scores of target gene expression were calculated as previously described (38). To compare ltNK cells and CD8^+^ tissue-resident memory T cells isolated from spleen and lung, the normalized counts dataset of GSE94964 was downloaded (25). A log2 normalization was applied and genes with a mean log2 value < 2 were removed. Subsequently, the log2 FC was calculated of genes expressed in both T cells and NK cells. Plots were generated by use of the ggplot2 package (35).

### Statistical Tests

To compare pairwise protein expression data or combined *Z* scores between ltNK, CD56^bright^, and CD56^dim^ NK cells, one-way ANOVA test was applied. Tukey’s correction was applied to correct for multiple testing. CD69^+^ and CD69^−^ memory T cells were compared using a paired *t*-test. *P*-values below 0.05 were considered as statistically significant. Statistics and scatter dot plots were generated using GraphPad Prism software (v7.00, Graphpad, La Jolla, CA, USA). Statistics of the RNA sequencing were calculated as described under “RNA sequence.”

## Results

### CD56^bright^, CD56^dim^, and ltNK Cells Constitute Three Distinct NK Cell Populations

Previously, we reported that CD69^+^CXCR6^+^ lymphoid tissue (lt)NK cells from BM, spleen, and lymph node are phenotypically and functionally distinct from circulating CD56^bright^ and CD56^dim^ NK cells (12). To find clues on the function and development of ltNK cells, we compared the transcriptional signatures of ltNK cells and the two conventional circulating NK cell subsets. From this point onward, the term ltNK cells refers to BM ltNK cells. NK cells from BM and blood of four healthy donors were first enriched using magnetic beads. Subsequently, NK cell populations were FACS-purified (from BM: 1. CD56^bright^, 2. CD56^dim^, 3. ltNK and from blood: 4. CD56^bright^, 5. CD56^dim^). LtNK cells were selected based on combined expression of CD69, CXCR6, and CD54 (Figure 1A). The blood-derived CD56^dim^ and BM-derived ltNK cells were initially separated into a NKG2A^+^ and NKG2A^−^ fraction (Figure 1A). When comparing these fractions, only three genes (of 9,382 genes included) had a significantly differential expression (Figures 1B,C). Therefore, we pooled the expression data proportionally from the NKG2A^+^ and NKG2A^−^ fractions in subsequent analyses.

We first evaluated differences in gene expression levels between the ltNK cells and the conventional NK (non-ltNK) cells within the BM. In ltNK cells, 674 genes were significantly upregulated while 679 genes were downregulated compared with CD56^bright^ and/or CD56^dim^ NK cells (Figure 1B). LtNK cells expressed 152 genes at the highest level and 194 at the lowest level of all NK cell subsets (Figure 1B). Moreover, unsupervised hierarchical clustering of the individual samples, based on expression of all 9,382 genes, demonstrated that the ltNK cells clustered (Figure 1D). Considering the high number of differentially expressed genes and the cluster formed by ltNK cells, the ltNK cells truly represent a distinct subset, rather than just resembling one of the circulating populations transiently trafficking through lymphoid tissues.

Conventional NK cell subsets in both lymphoid tissues and blood are phenotypically similar (12). This prompted us to hypothesize that the CD56^bright^ and CD56^dim^ NK cells in BM represent the circulating NK cells. Indeed, the transcriptome analysis revealed that only 16 and 76 genes of the total 9,382 genes were significantly differentially expressed in marrow derived versus circulating CD56^dim^ and CD56^bright^ NK cells, respectively. Correspondingly, circulating CD56^dim^ and CD56^bright^ NK cells cluster together with the BM derived CD56^dim^ and CD56^bright^ NK cells, respectively (Figure 1D). Comparison of the CD56^dim^ with CD56^bright^ NK cells from blood revealed that 1,181 (647 + 534) of the 9,382 genes were differentially expressed (Figure 1C). Comparing the same populations in BM yielded 720 (364 + 356) genes of which 74% overlapped with the differentially expressed genes in blood (Figure 1C). Despite the inevitable blood contamination of BM aspirates, our findings suggest that CD56^bright^ and CD56^dim^ NK cells are two distinct subsets, which do not differ between blood and marrow.

### LtNK Cells Are Eomes^high^Tbet^low^

**Figure 2:**
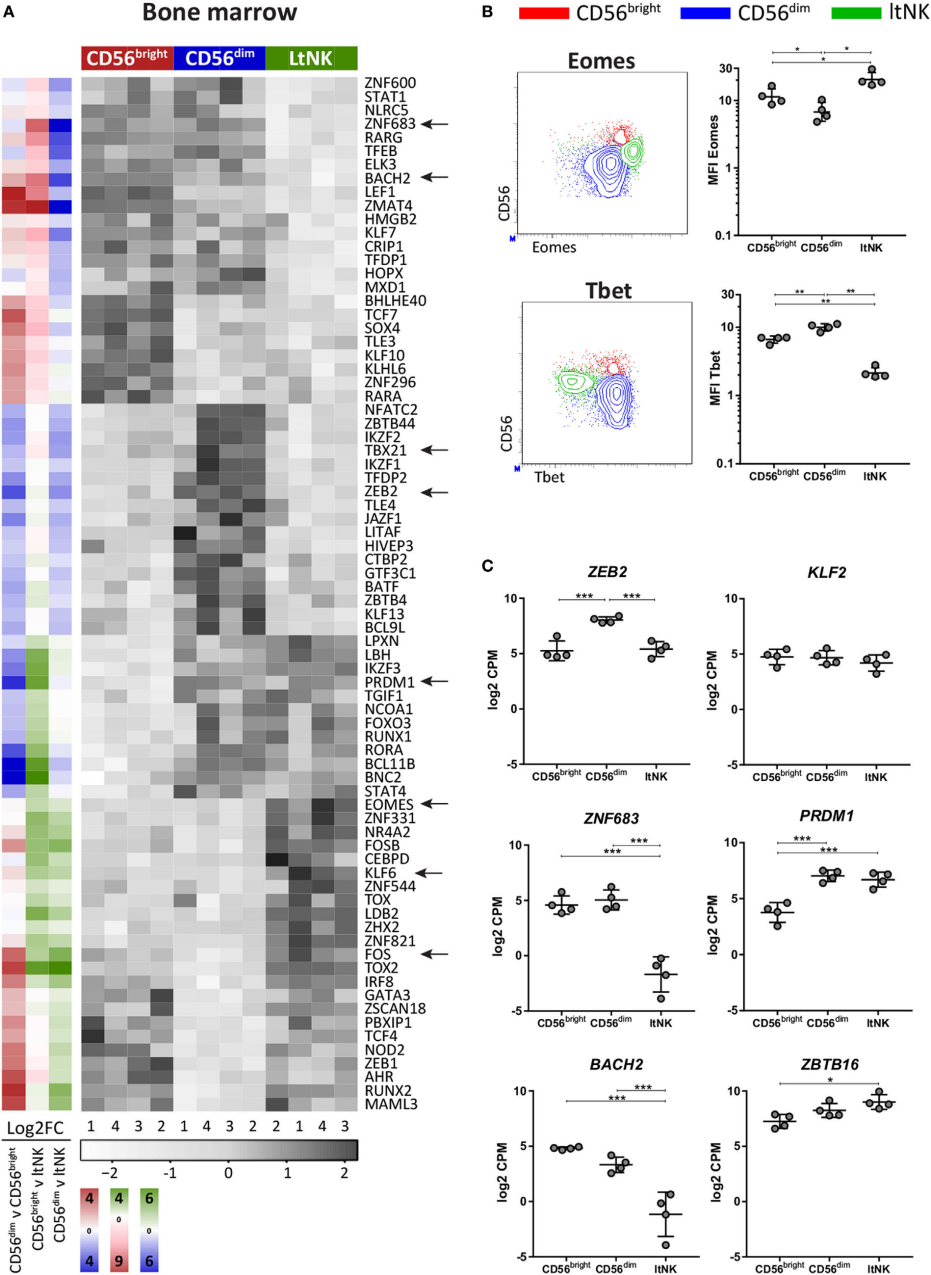
LtNK cells are Eomes^high^Tbet^low^. **(A)** Heatmap illustrates normalized mRNA expression values of transcription factors, which have the highest or lowest mRNA expression [false discovery rate (FDR) <0.05] in 1 of the 3 bone marrow (BM)-derived natural killer (NK) cell subsets. The column side bars represent the log2-fold change (FC) of gene expression levels in one NK cell subset versus another. The color indicates in which NK cell population the gene is expressed at the highest level (green = ltNK, red = CD56^bright^, blue = CD56^dim^). The color intensity represents the magnitude of the FC. **(B)** Eomes and Tbet expression of CD56^bright^ (red), CD56^dim^ (blue), and ltNK cells (green), as determined by flow cytometry. Shown are representative dot plots of BM-derived NK cells. MFI, mean fluorescence intensity. *P < 0.05, **P < 0.01, by one-way ANOVA. **(C)** Normalized mRNA expression levels of selected transcription factors. *FDR < 0.05, ***FDR < 0.001. CPM, counts per million of uniquely mapped reads. FDR, false discovery rate. Error bars represent mean ± SD in **(B,C)**.

To evaluate whether the transcriptional network differs between the NK cell subsets, transcription factor profiles of the NK cell populations in BM (Figure 2A) and blood were generated (Figure S2 in Supplementary Material). *EOMES* and *TBX21* (Tbet) were the highest and lowest expressed by ltNK cells, respectively (Figure 2A). In line with this, ltNK cells had an Eomes^high^Tbet^low^ phenotype. Eomes is often used to discriminate NK cells (Eomes^+^) from the helper innate lymphoid cells (Eomes^−^), confirming that ltNK cells belong to the NK cell lineage (Figure 2B) (39). Human liver-resident CXCR6^+^ NK cells were previously found to be Eomes^high^Tbet^low^ as well (8, 40). In both murine and human NK cells, *ZEB2* transcript levels increase during the process of NK cell maturation (41). mRNA levels of *ZEB2* in ltNK cells were equal to CD56^bright^ NK cells and lower than in CD56^dim^ NK cells (Figure 2C).

Several transcription factors are associated with development of tissue-resident lymphocytes. Because Kruppel-like factor 2 (KLF2) upregulates S1PR1 and CD62L expression, downregulation of KLF2 supports tissue-residency (42, 43). We found only a minor non-significant difference in *KLF2* expression between ltNK cells and circulating NK cells (Figure 2C). Maintenance of murine liver-resident NK cells is dependent on *Zfp683* (Hobit) while maintenance of conventional NK cells is not (26). This contradicts human NK cells: *ZNF683* (HOBIT*)* was expressed at lower levels in ltNK cells, while higher levels were observed in CD56^bright^ and CD56^dim^ NK cells (Figure 2C), as was previously shown by flow cytometry on the latter two populations from blood (44). *PRDM1* (Blimp1), which regulates maintenance of murine tissue-resident T cells did not differ in expression between ltNK and CD56^dim^ NK cells (Figure 2C) (26). However, the transcriptional repressor of PRDM1, *BACH2*, was lower expressed in ltNK cells compared to the circulating NK cells, while *ZBTB16*, which encodes PLZF, a repressor of BACH2, was elevated (Figure 2C) (45, 46).

### Adhesion Molecule Profile of ltNK Cells Reveals Tissue-Resident Features

**Figure 3:**
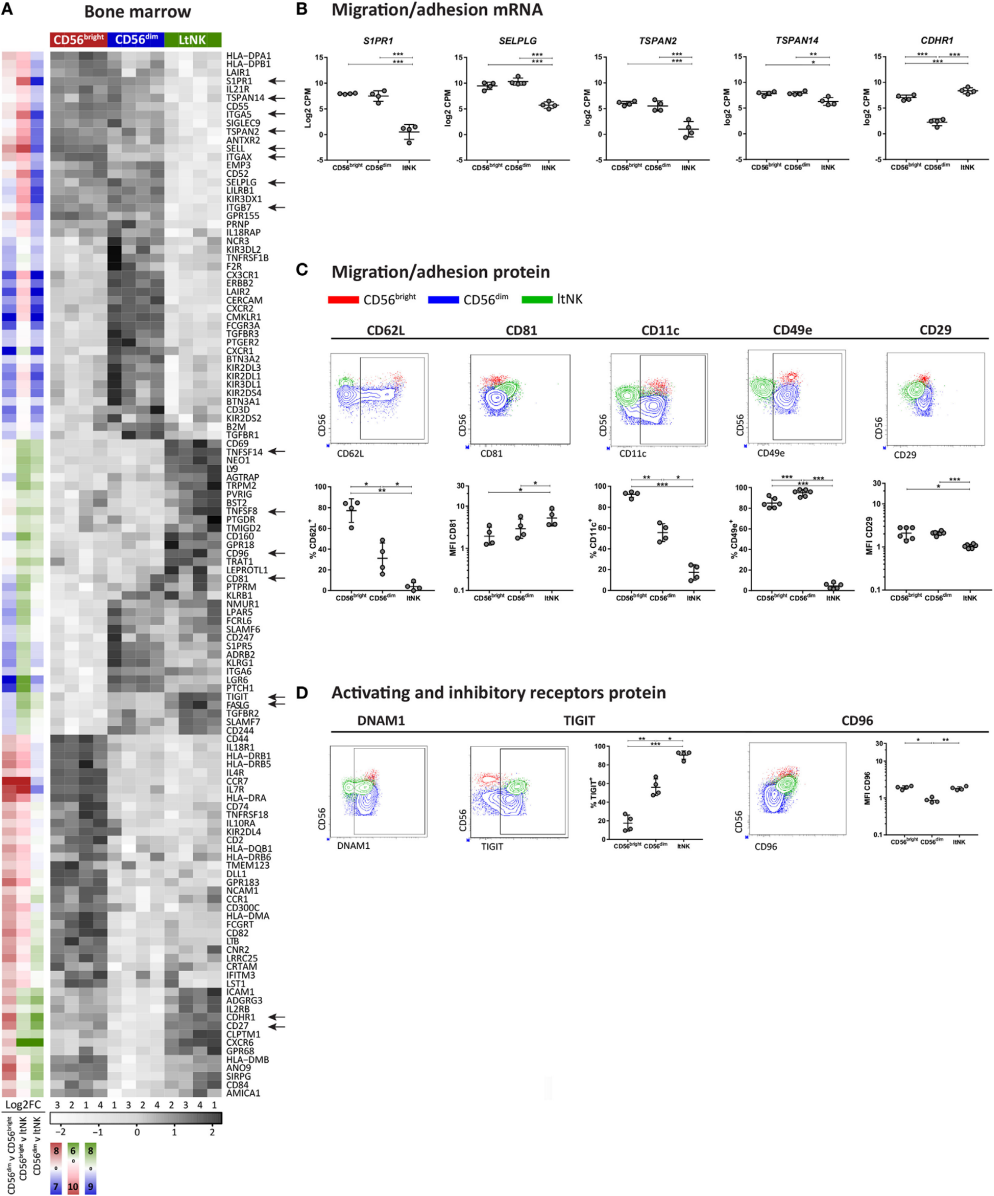
Surface molecule profile of ltNK cells reveals altered adhesion molecule repertoire. **(A)** Heatmap shows scaled mRNA expression values of genes, which encode surface molecules and have the highest or lowest mRNA expression [false discovery rate (FDR) < 0.05] in 1 of the 3 bone marrow (BM)-derived natural killer (NK) cell subsets. The column side bars represent the log2-fold change (FC) of gene expression levels in one NK cell subset versus another. The color indicates in which NK cell population the gene is expressed at the highest level (green = ltNK, red = CD56^bright^, blue = CD56^dim^). The color intensity represents the magnitude of the FC. **(B)** Normalized mRNA expression levels of differentially expressed surface molecules involved in migration/adhesion. *FDR < 0.05, **FDR < 0.01, ***FDR < 0.001. CPM, counts per million of uniquely mapped reads; FDR, false discovery rate. **(C,D)** Validation of mRNA expression of surface molecules by flow cytometry. Shown are representative dot plots of BM-derived NK cells. MFI, mean fluorescence intensity. *P < 0.05, **P < 0.01, ***P < 0.001, by one-way ANOVA. Mean ± SD are shown in **(B–D)**.

To obtain clues on environmental interactions mediated by ltNK cells, we analyzed the differentially expressed genes encoding surface molecules and visualized the result in heatmaps (Figure 3A; Figure S2 in Supplementary Material). Among the surface molecules up- and downregulated in ltNK cells compared to circulating NK cells, we identified several molecules associated with tissue-residency. *S1PR1*, the receptor for S1P, which promotes tissue egress was expressed at the lowest mRNA level of all NK cell subsets (Figures 3A,B). *SELPLG* (CD162, Selectin P ligand) and *SELL* (CD62L, l-selectin), both involved in lymphocyte recruitment from blood to tissues *via* interaction with vessel endothelium, were downregulated as well (Figures 3A,B). By flow cytometry, we confirmed that CD62L was not expressed by ltNK cells (Figure 3C).

Other adhesion molecules in the list of differentially expressed surface molecules were the tetraspanins *TSPAN2*↓, *TSPAN14*↓, and *CD81↑* (TSPAN28), the integrins *ITGAX*↓ (CD11c), *ITGB7*↓, and *ITGA5*↓ (CD49e), and the cadherin *CDHR1↑* (Figures 3A,B). Consistent with the RNA sequence data, CD81 protein was expressed at a higher intensity on the cell surface of ltNK cells compared with the conventional NK cells (Figure 3C). CD11c expression was indeed significantly lower on ltNK cells compared with CD56^bright^ and CD56^dim^ NK cells. Expression of ITGB7 did not differ between ltNK and circulating NK cells (Figure S3A in Supplementary Material). Remarkably, while the vast majority of conventional NK cells expressed CD49e, ltNK cells lacked this integrin on their surface (Figure 3C). The dimerizing partner of CD49e, ITGB1 (CD29) was also expressed at a significantly lower intensity on ltNK cells compared with the conventional NK cells (Figure 3C). Overall, the adhesion molecule profile of ltNK cells differs significantly from both the CD56^bright^ and CD56^dim^ NK cells.

### LtNK Cells Express the Inhibitory Receptors TIGIT and CD96 and Lack Cytolytic Proteins

**Figure 4:**
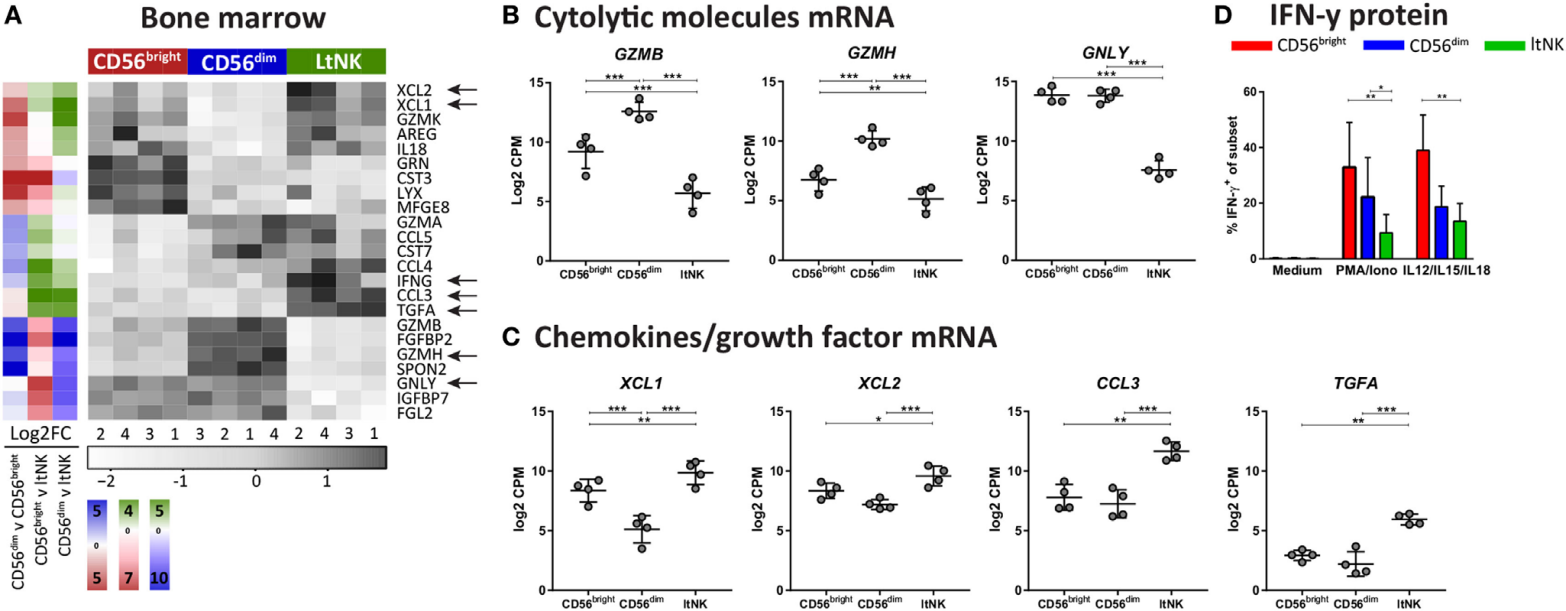
Effector molecule profile of ltNK cells highlights low killing capacity. **(A)** Heatmap depicts normalized mRNA expression levels of genes, which encode effector molecules and have the highest or lowest expression in 1 of the 3 bone marrow (BM) derived natural killer (NK) cell subsets [false discovery rate (FDR) < 0.05]. The column side bars represent the log2-fold change (FC) of gene expression levels in one NK cell subset versus another. The color indicates in which NK cell population the gene is expressed at the highest level (green = ltNK, red = CD56^bright^, blue = CD56^dim^). **(B,C)** Normalized mRNA expression values of differentially expressed **(B)** cytolytic molecules (granzyme B, granzyme H, and granulysin) and **(C)** chemokines (XCL1, XCL2, CCL3) and the growth factor TGFα. CPM, counts per million of uniquely mapped reads. *FDR < 0.05, **FDR < 0.01, ***FDR < 0.001. FDR, false discovery rate. **(D)** Intracellular IFN-γ expression of CD56^bright^, CD56^dim^, and ltNK cells. BM mononuclear cells (*n* = 7) were stimulated for 4 h with PMA/ionomycin or the combination of IL12, IL15, and IL18. After 1 h, Golgistop was added to the culture. *P < 0.05, **P < 0.01, by one-way ANOVA. Mean ± SD are shown in **(B–D)**.

Natural killer cell effector function depends on signals derived from activating and inhibitory receptors and on the potential of NK cells to modulate other cells by expressing ligands or secrete effector molecules. Genes encoding TNF superfamily members were highly expressed by ltNK cells: *CD27, TNFSF14* (LIGHT), *TNFSF8 (*CD30L*)*, and *FASLG* (Figure 3A). Earlier, we demonstrated the high protein expression of CD27 on ltNK cells (12). However, LIGHT and CD30L were not detected on the surface of resting NK cells (Figure S3B in Supplementary Material). Although PMA/ionomycin stimulation induced LIGHT expression, mainly on the CD56^bright^ and ltNK cell subset, CD30L expression could not be induced (Figure S3B in Supplementary Material). In addition, the surface molecule transcriptome of ltNK cells contained various receptors, which are known to inhibit NK cell activity. *TIGIT* and *CD96* are inhibitory receptors, which engage the same ligands as the stimulatory receptor DNAM1 (*CD226*) and were expressed at high levels in ltNK cells (Figure 3A). DNAM1 is expressed by 40% of the ltNK cells, while all conventional NK cells express DNAM1 (Figure 3D) (12). Flow cytometry confirmed that nearly 100% of ltNK cells express TIGIT, in contrast to CD56^dim^ (mean 56%) and CD56^bright^ (mean 18%) NK cells (Figure 3D). The CD96 expression of ltNK cells was comparable to the CD56^bright^ NK cell population (Figure 3D). In line with the inhibitory receptor repertoire, we previously reported that ltNK cells do not efficiently lyse K562 target cells (12). Furthermore, the genes encoding the cytolytic molecules granzyme B (*GZMB*), granzyme H (*GZMH*), and granulysin (*GNLY*) were expressed at the lowest level by ltNK cells (Figures 4A,B). At the protein level, resting ltNK cells did not express granzyme B, but did express perforin albeit at a lower level than CD56^dim^ NK cells (12). Collectively, ltNK cells express the inhibitory receptors TIGIT and CD96 and are not endowed with a full cytotoxic machinery.

### High Transcript Levels of *IFNG* in ltNK Cells Cannot Be Recapitulated at the Protein Level *In Vitro*

Potentially, other secreted effector molecules are involved in ltNK cell functioning. Elevated transcript levels of XCL1, XCL2, CCL3, and TGFα were detected in ltNK cells (Figures 4A,C). Strikingly, *IFNG* mRNA was also enriched in ltNK cells. This suggests that ltNK cells can rapidly modulate the immune response by producing IFN-γ upon triggering, as was earlier demonstrated for CD8^+^ tissue-resident memory T cells in spleen and lung (23, 25). However, we were not able to activate IFN-γ production in ltNK cells. Strong short-term stimulation with PMA/ionomycin or IL12/IL15/IL18 did not result in a high percentage of IFN-γ producing ltNK cells (Figure 4D). Therefore, triggering of IFN-γ production in ltNK cells may require different stimuli than classically used for CD56^bright^ and CD56^dim^ NK cells.

### Gene Set Enrichment Analysis (GSEA) Reveals Potential Inducers of ltNK Cell Transcriptome

**Figure 5:**
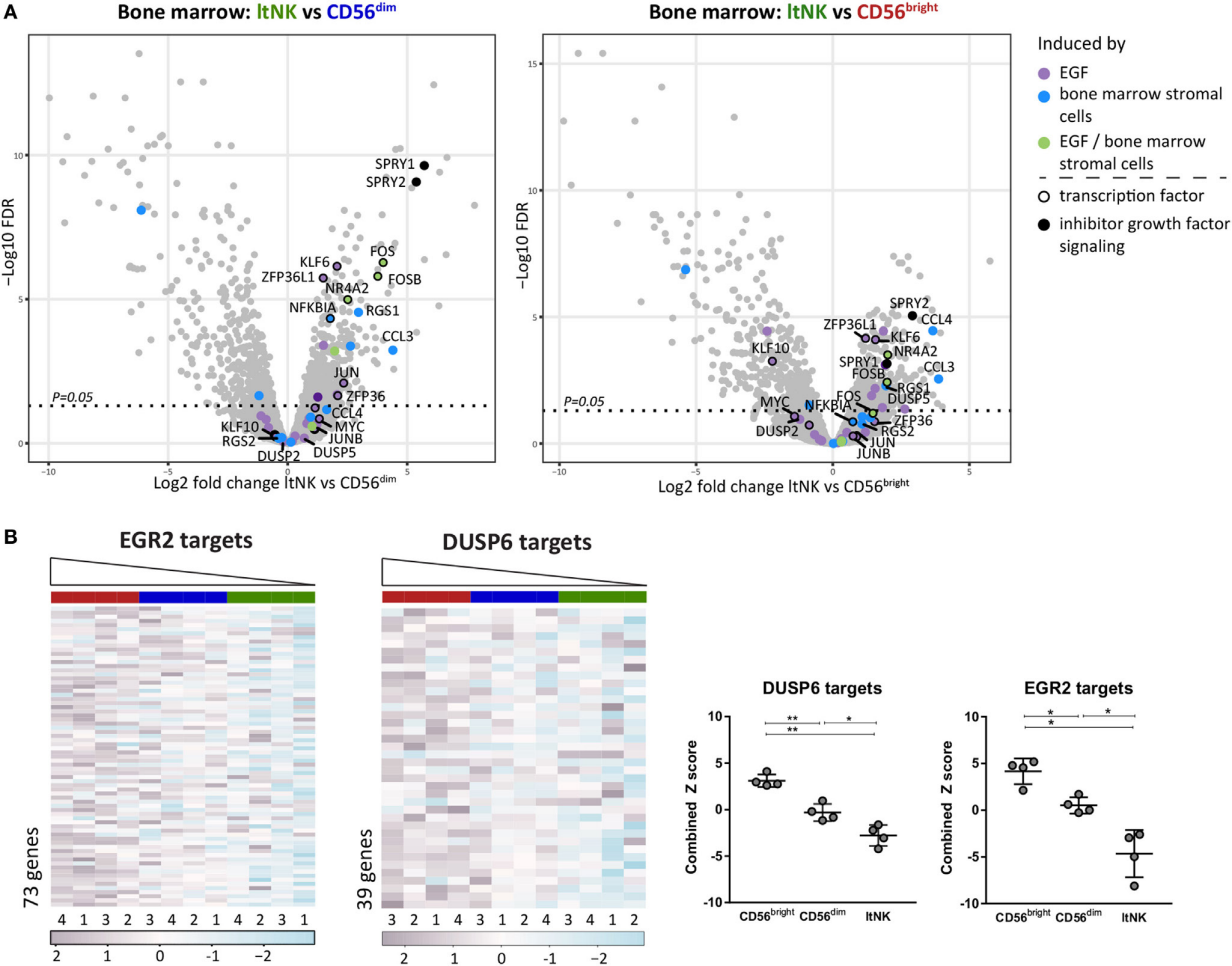
Gene set enrichment analysis (GSEA) reveals potential inducers of ltNK cell transcriptome. **(A)** The gene sets epidermal growth factor (EGF) signaling (M16311) and bone marrow (BM) stimulation (M5929) were enriched in ltNK cells, as identified by GSEA using CAMERA and the Broad institute gene set collections. The log2 fold change of ltNK versus CD56^bright^ or ltNK versus CD56^dim^ is plotted against the false discovery rate (FDR) in volcanoplots. Genes that were induced by either EGF, BM stromal cells, or both are depicted. SPRY1 and SPRY2 are two inhibitors of growth factor signaling. FDR, false discovery rate. **(B)** GSEA revealed that targets that are repressed by EGR2 (M12804) or DUSP6 (M7339) are downregulated in ltNK cells. Heatmaps show the normalized expression values of the corresponding genes, which are repressed by EGR2 (*n* = 73) and DUSP6 (*n* = 39). The column order is based on the combined *Z* score of each donor natural killer cell population (high → low). The combined *Z* score is a quantification of the overall expression level of the target genes. Error bars represent mean ± SD. *P < 0.05, **P < 0.01, by one-way ANOVA.

To obtain clues on over- or underrepresented pathways in ltNK cells, we performed a GSEA by use of CAMERA and the Broad institute gene set collections. In addition, GSEA can uncover molecules, which might induce the genetic program of ltNK cells by analyzing expression of the downstream target genes. Only gene sets, which were significantly up- or downregulated in ltNK cells compared to both CD56^bright^ and CD56^dim^ NK cells (FDR < 0.05) were further analyzed. We first selected the gene sets, which were upregulated in ltNK cells versus the circulating NK cells (Table S4A in Supplementary Material). Among those gene sets, we identified epidermal growth factor (EGF) signaling and BM stromal cell stimulation (Figure 5A). EGF has been shown to induce a network of transcription factors, including NR4A2, FOS, JUN, KLF6, and ZFP63L1, which were all expressed the highest by ltNK cells (Figure 2A) (47). Moreover, SPRY1 and SPRY2, two inhibitors of EGF and other growth factor receptor tyrosine kinase signaling were also enriched in ltNK cells, which is suggestive of an inhibitory feedback loop (48). These results favor the hypothesis of a growth factor induced transcriptional program in ltNK cells, potentially mediated by cells from the BM microenvironment.

Second, we selected the downregulated gene sets, which included many transcription factors (Table S4B in Supplementary Material). Among the transcription factor gene sets, three downregulated gene sets included genes, which expression was negatively affected by the responsible transcription factor, indicating that the corresponding transcription factors are more active in ltNK cells: EGR2, BCL3, and YBX1. The expression of the target genes of these three transcription factors was indeed the lowest in the ltNK subset, as quantified by the combined *Z* score (Figure 5B; Figure S4 in Supplementary Material). EGR2 was earlier reported to be higher expressed by lung-resident CD8^+^ memory T cells than by circulating effector memory CD8^+^ T cells (23). In addition, another set of genes, which expression is inhibited by the dual-specificity phosphatase DUSP6 (inhibitor of MAP kinase signaling), were downregulated in ltNK cells (M7339, Figure 5B). Recently, DUSP6 has been identified as a core tissue-resident memory T cell molecule (25). These results imply that EGR2 and DUSP6 are candidate molecules involved in induction or maintenance of ltNK cells.

### LtNK Cells Have Less Proliferative Capacity Than CD56^bright^ and CD56^dim^ NK Cells

**Figure 6:**
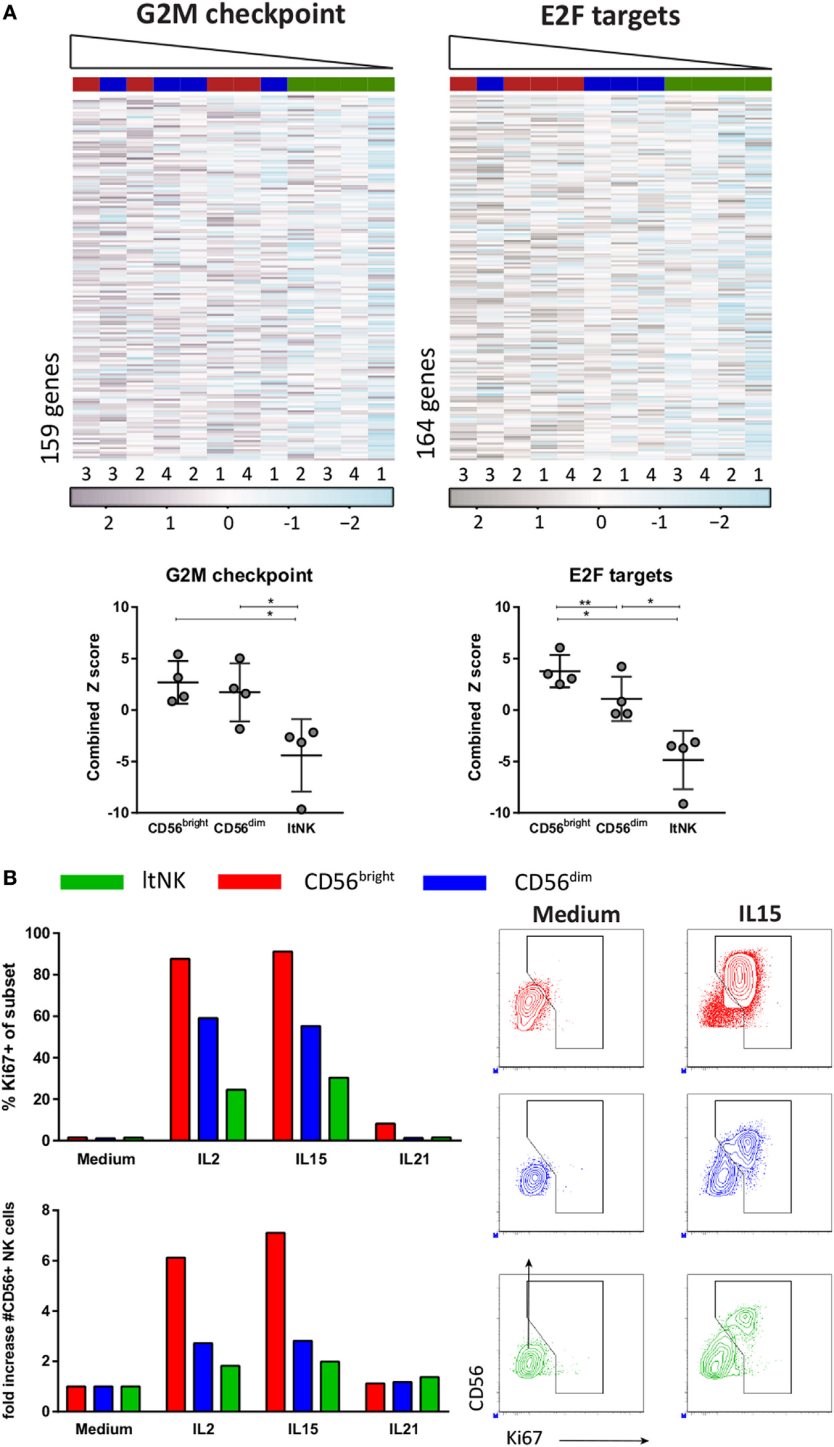
Proliferative capacity of ltNK cells is lower compared with CD56^bright^ and CD56^dim^ natural killer (NK) cells. **(A)** Gene set enrichment analysis using CAMERA and the Broad institute gene collections revealed that many cell-cycle related genes are downregulated by ltNK cells. Heatmaps show the normalized expression values of the hallmark gene sets G2M checkpoint (M5901) and E2F targets (M5925). The column order is based on the combined *Z* score of each donor NK cell population (high → low). The combined *Z* score is a quantification of the overall expression level of the genes per donor derived NK cell population. *P < 0.05, **P < 0.01, by one-way ANOVA. Shown is mean ± SD. **(B)** Proliferative capacity was assessed by stimulating sorted bone marrow-derived NK cell populations for 6 days with IL2, IL15, or IL21. Intracellular Ki67 expression was measured by flow cytometry. The number of CD56^+^ NK cells is based on flow cytometer counts. Shown are dot plots of medium control and IL15 stimulated NK cell populations.

The downregulated gene sets of the GSEA also included many cell-cycle related genes (Table S4C in Supplementary Material). The hallmark data set “G2M checkpoint” represents genes involved in progression through the cell division cycle. Expression of those genes was the lowest in ltNK cells and the highest in CD56^bright^ NK cells (Figure 6A). The same was true for expression of the targets of E2F (“Hallmark E2F targets”), a family of transcription factors, which regulates the cell cycle (Figure 6A). To assess their proliferative capacity, we sorted ltNK, CD56^bright^, and CD56^dim^ NK cells from BM and stimulated the cells with IL2, IL15, or IL21 (Figure 6B). In agreement, ltNK cells had the lowest expansion and lowest intracellular Ki67 expression upon stimulation with IL2 and IL15. As previously described, the CD56^bright^ NK cells were highly proliferative (49).

### LtNK and Tissue-Resident CD8^+^ Memory T Cells Share a Core Transcriptional and Phenotypic Profile

**Figure 7:**
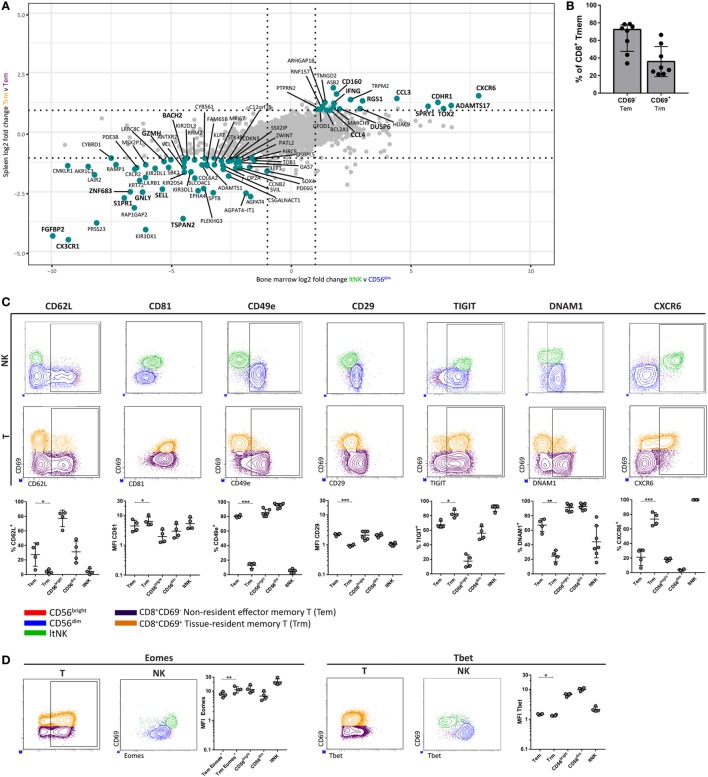
LtNK cells and tissue-resident CD8^+^ memory T cells share a core transcriptional and phenotypic profile. **(A)** The datasest GSE94964 contains RNAseq data of spleen-derived CD8^+^ effector memory T cells (CD3^+^CD8^+^CD45RA^−^CCR7^−^), which were subdivided into CD69^+^ tissue-resident memory (Trm) and CD69^−^ effector memory T (Tem) cells. We compared this gene set to our RNAseq data to determine a core gene signature among Trm and ltNK cells in lymphoid tissues. The log2-fold change (FC) of 8,700 genes [which were both expressed in T and natural killer (NK) cells] was calculated. The log2 FC of ltNK versus CD56^dim^ was plotted against the log2 FC of CD8^+^ Trm against Tem cells. Figure S5 in Supplementary Material depicts the plot, which contains the log2 FC of ltNK versus CD56^bright^ NK cells. Core resident genes, which are depicted in turquoise were identified as genes with a log2 FC ≥ 1 or ≤−1 in all three comparisons. **(B)** Frequency of CD69^−^ non-resident (Tem) and CD69^+^ resident CD8^+^ memory T (Trm) cells in bone marrow (BM). CD8^+^ memory T cells were defined as CD3^+^CD8^+^CCR7^+^ and included both effector memory and end-stage effector memory T cells. **(C,D)** Protein expression of surface molecules and transcription factors, which were differentially expressed by ltNK versus circulating NK cells were determined on CD8^+^ memory T cells (CD69^+^ versus CD69^−^). For comparison, plots visualizing the ltNK, CD56^bright^, and CD56^dim^ NK cells are shown. The Eomes MFI on the CD8^+^ memory T cells was determined only on the Eomes^+^ CD8^+^ memory T cells, rather than on the whole population (as was done for the NK cells). Statistics of NK cell protein expression comparisons are shown in Figure 3. Dot plots depict a representative BM donor. MFI, mean fluorescence intensity. *P < 0.05, **P < 0.01, ***P < 0.001, by two-tailed paired *t*-test. Median and interquartile range are shown in **(B)**. Mean ± SD are shown in **(C,D)**.

Many studies transcriptionally profiled CD8^+^ tissue-resident memory T (Trm) cells from various tissues. As earlier stated, *DUSP6* and *EGR2*, identified in our GSEA, were reported to be higher expressed in human CD8^+^ Trm cells compared to circulating counterparts (23, 25). This led us to hypothesize about a transcriptional program, which is shared between ltNK and CD8^+^ Trm cells. We aimed to perform a comparative analysis of RNA sequence data from spleen derived CD69^+^CD8^+^ Trm and CD69^−^CD8^+^ effector memory T (Tem) cells (25), and our own NK cell dataset. We selected genes expressed in both T and NK cells and plotted the log2 FC of CD8^+^ Trm versus Tem cells against the log2 FC of ltNK versus CD56^dim^ NK cells, and ltNK versus CD56^bright^ NK cells (Figure 7A; Figure S5 in Supplementary Material). Importantly, the most differentially expressed genes were up- or downregulated in the same direction in all comparisons. Core-resident genes were selected based on a log2 FC ≥ 1 or ≤−1 in all three comparisons. In total, we revealed 83 genes, which have a universal differential expression pattern among resident CD8^+^ memory T and NK cells (Table S5 in Supplementary Material). Among the core genes, we identified *S1PR1*↓, *CD62L*↓, *DUSP6↑, CXCR6↑, MKI67*↓, and *IFNG↑*. This prompted us to investigate whether the surface molecule and transcription factor profile of ltNK cells (CD62L^−^, CD81^high^, CD11c^−^, CD49e^−^, CD29^low^, TIGIT^+^, DNAM1^low^, CXCR6^+^, Eomes^high^, Tbet^low^) is similar to CD8^+^ Trm cells. Although CD69^+^ memory T cells comprise a highly diverse subset across tissues, CD69 was shown to be the major marker delineating circulating from resident T cells (25, 50). For this analysis, we compared resident CD69^+^ (Trm) and non-resident CD69^−^ (Tem) CD8^+^ memory T cells from human BM (Figure S1 in Supplementary Material). Trm cells represented 28% (median) of BM CD8^+^ memory T cells (Figure 7B). In contrast to Trm cells from the often evaluated mucosal and epithelial tissues, CD103 was hardly expressed by CD8^+^ Trm cells in the BM (Figure S6 in Supplementary Material). Unlike NK cells, CD8^+^ memory T cells universally lacked CD11c (Figure S6 in Supplementary Material). Like ltNK cells, CD8^+^ Trm cells were CD62L^−^, CD81^high^, CD49e^−^, CD29^low^, TIGIT^+^, and DNAM^−^. Moreover, the majority of CD8^+^ Trm cells expressed CXCR6 (Figure 7C). In addition, Eomes and Tbet were higher and lower expressed in CD8^+^ Trm compared to CD8^+^ Tem cells in BM (Figure 7D). Collectively, we defined a core gene and molecular signature, which characterizes both lymphoid tissue-derived ltNK cells and CD8^+^ Trm cells.

## Discussion

The majority of NK cell studies focused on circulating NK cells, which resulted in the classical subdivision of NK cells in the CD56^bright^ and CD56^dim^ NK cell subsets. We recently defined a non-circulating NK cell population, based on the combined expression of CD69 and CXCR6, which resides in lymphoid tissues. In this study, we further analyzed the CD69^+^CXCR6^+^ ltNK cells and performed RNA sequence analysis to provide more insight into the biology of ltNK cells. We demonstrated that the surface receptor, adhesion molecule, and transcription factor profiles of ltNK cells significantly differ from that of circulating NK cells. Thereby, we confirmed that ltNK cells constitute a unique subset and not reflect one of the two circulating populations trafficking through tissues, transiently acquiring a different phenotype. Additionally, we defined a core gene signature and surface molecule profile, which is shared by ltNK cells and CD8^+^ Trm cells, thereby further specifying tissue-residency.

The transcriptome and phenotype of ltNK cells support the notion that ltNK cells are tissue-resident. Molecules involved in cell trafficking are downregulated in both tissue-resident murine NK cells, human liver-resident NK cells, and ltNK cells: i.e., *S1PR1*, CCR7, and CD62L (8, 9, 12, 51, 52). S1PR1 enables tissue egress, while CCR7 and CD62L are required to enter lymphoid tissues *via* high endothelial venules (4, 5, 53). Several transcription factors are known to regulate expression of these trafficking molecules, mainly deduced from murine cell lineage specific knock-out models. The transcription factor (KLF2) promotes *S1pr1* and *Sell* (CD62L) expression (42, 43). However, *KLF2* was neither significantly downregulated by ltNK cells nor by human liver-resident NK cells (42, 43). *Hobit* represses *S1pr1, Klf2*, and *Ccr7* and is preferentially expressed in murine-resident lymphocytes (26). However, ltNK cells show profoundly reduced *HOBIT* expression, whereas the circulating NK cells express high levels. Hence, despite the compatible surface profile of migration/adhesion molecules, the transcriptional network underlying tissue-residency in human ltNK cells seems to differ from that in mice.

The comparative gene expression analysis revealed several interesting differences in the integrin and tetraspanin repertoire between ltNK and circulating NK cells. By flow cytometry, we confirmed that these differences were also present at the protein level. Compared to circulating NK cells, CD81 (TSPAN28) and CD11c (ITGAX) were found to be higher and lower expressed by ltNK cells, respectively. Importantly, we identified the lack of CD49e (ITGA5) as a key marker to delineate ltNK cells from circulating NK cells. In line with this, ltNK cells had lower expression of the dimerizing partner CD29 (ITGB1). A recent paper demonstrated that human liver-resident NK cells are also negative for CD49e (10). Moreover, murine tissue-resident NK cells are marked by absence of CD49b, indicating that an altered integrin repertoire is characteristic for resident NK cells (51). Collectively, both the absence of CD49e and the presence of CD69 and CXCR6 can be used to define resident ltNK cells in humans.

In addition to the adhesion molecule profile, we observed that the inhibitory receptors TIGIT and CD96 were highly expressed by ltNK cells. TIGIT and CD96 share ligands with the activating receptor DNAM1 (CD226), which is expressed by the minority of ltNK cells (54). Engagement of TIGIT by PVR (CD155) or PVRL2 (CD112) can inhibit human NK cell cytotoxicity (55). Furthermore, the expression of TIGIT is inversely correlated with IFN-γ production (56). The functional profile of ltNK cells indeed revealed reduced expression of cytotoxic molecules (PRF1, GZMB, *GZMH, GNLY*), as well as reduced capacity to kill, to proliferate, and to produce IFN-γ, despite high levels of *IFNG* mRNA (12). This functional profile suggests that the effector function of ltNK cells is restrained. Alternatively, the appropriate stimuli to engage the functional potential of ltNK cells have not yet been identified.

By performing GSEA, we identified the transcription factor EGR2, the phosphatase DUSP6, the growth factor EGF, and “bone marrow stromal cells” as potential regulators of the ltNK cells transcriptome. EGR2 has been identified as transcriptional activator of *Zbtb16* (PLZF) (57). PLZF represses *Bach2*, which on its turn represses *Prdm1* (Blimp1) (45, 46). In line with this axis, *ZBTB16* was the highest expressed in ltNK cells, while *BACH2* was barely expressed at mRNA level in ltNK cells. LtNK cells express *PRDM1* at levels comparable to CD56^dim^ NK cells but higher than CD56^bright^ NK cells. These findings are supportive for a regulatory axis with EGR2 as a central regulator. DUSP6 is an inhibitor of MAP kinase signaling. Although the function of DUSP6 in NK cells has not been reported, high levels of DUSP6 are associated with reduced TCR sensitivity (58). Moreover, DUSP6 inhibits TCR signaling and IFN-γ production in murine CD4 T cells, revealing another potential explanation for the difficulty we encountered in evoking IFN-γ production in ltNK cells (59, 60). Finally, genes that are upregulated by EGF and BM stromal cells were enriched in ltNK cells. Notably, EGF induces *EGR2* as well (61). Therefore, it is tempting to speculate that growth factors secreted by stromal cells induce the ltNK cell transcriptome.

DUSP6 and EGR2 were reported to be differentially expressed by human CD8^+^ Trm cells versus their circulating counterparts (23, 25). This finding led us to speculate about a transcriptional program, which is shared among resident lymphocytes. We identified a core gene and surface molecule signature among ltNK cells and lymphoid tissue derived CD8^+^ Trm cells, which includes molecules associated with tissue-residency (*S1PR1*↓, CD62L↓), adhesion molecules (CD49e↓, CD29↓, CD81↑, *TSPAN2*↓), chemokine receptors (CXCR6↑, *CX3CR1*↓, *CXCR2*↓), activating/inhibitory receptors (TIGIT↑, DNAM1↓), transcription factors (Eomes↑, Tbet↓, *HOBIT*↓, *BACH2*↓), effector molecules (*IFNG↑, CCL3↑, FGFBP2*↓, *GNLY*↓, *GZMH*↓), and regulatory molecules (*DUSP6↑, RGS1↑*, Ki67↓). Notably, human liver-resident NK cells exhibit strong similarities to ltNK cells regarding phenotype (CD49e^−^CXCR6^+^Eomes^high^Tbet^low^) as well as the aforementioned core gene signature (except for *CCL3*) (7–10, 40). Moreover, human hepatic CD8^+^ Trm cells are CXCR6^+^Eomes^high^Tbet^low^ and have reduced mRNA levels of *HOBIT* (24). These findings define a core gene signature of residency, which is shared by human tissue-resident NK cells and CD8^+^ Trm cells in lymphoid tissues and likely also in liver.

It is important to note that phenotypic heterogeneity does exist between tissue-resident lymphocytes in human non-mucosal tissue (e.g., spleen, BM, and liver) and in mucosal or epithelial tissues (e.g., tonsil, gut, skin, and lung), as has been elegantly demonstrated by mass cytometry (50). Although it is generally assumed that Trm cells are Eomes^−/low^ (30, 62), we demonstrated that BM CD8^+^ Trm cells are Eomes^high^. Potentially, Eomes expression is dependent on the tissue where the lymphocytes reside. In contrast to the Eomes^high^ BM derived ltNK cells and CD8^+^ Trm cells, human lung CD103^+^CD8^+^ Trm cells are Eomes^low^ and skin CD103^+^CD8^+^ Trm have reduced transcript levels of *EOMES* (21, 23). Moreover, CD49a and CD103 are both highly expressed by human tissue-resident NK cells in tonsil and CD8^+^ Trm cells in tonsil, lung, and skin, while those markers are nearly absent on ltNK cells and spleen and BM-derived CD8^+^ Trm cells (11, 12, 23, 50, 63). Eomes downregulation and CD103 and CD49a upregulation are hallmarks of TGF-β imprinting, indicating that local environmental cues govern the tissue-specific phenotype of tissue-resident lymphocytes (31, 64, 65). Therefore, it is important to distinguish non-mucosal lymphoid tissue from mucosal or epithelial tissue when discussing tissue-resident lymphocytes.

The function of ltNK cells still remains enigmatic. Taking all our observations into consideration, both CD8^+^ Trm cells and ltNK cells have low proliferative capacity (25), reduced expression of cytotoxic molecules, while expressing high *IFN-*γ mRNA. A key advantage of lymphocytes residing in tissues could be a rapid response to recurrent pathogens. For murine CD8^+^ Trm cells, it has been proven that pathogen-specific CD8^+^ Trm cells in skin can protect against and clear recurrent infections better than circulating memory CD8^+^ T cells (32, 66–70). Murine liver-resident NK cells confer hapten- and virus-specific memory responses (51, 71). These studies support a model in which ltNK cells and CD8^+^ Trm cells reside in lymphoid tissues awaiting to encounter a specific stimulus. As an alternative for the perforin/granzyme B-mediated pathway, cytolysis could involve death receptor ligands. FASL was upregulated in both ltNK cells and lung-resident CD8^+^ Trm cells (23). Still, the question remains which activating signals can release the functional brake or whether a distinct function is exerted.

The transcriptional signature of ltNK cells contributed to our understanding of the ltNK cell biology and yielded two important concepts. First, the ltNK cells are truly distinct from the two circulating NK cell subsets. Second, the transcriptional signature of the ltNK cells is very similar to that of (splenic) CD8^+^ Trm cells. Despite the lack of transcriptional data on simultaneously purified CD8^+^ Trm and Tem cells from BM in our study, we were able to provide further insight into tissue-resident features by comparing our own data with a publically available dataset. Validation of the BM ltNK and CD8^+^ Trm cell phenotype in other (lymphoid) tissues would be interesting, but is missing in this study due to the lack of healthy donor tissues. It remains presently unknown, which stimuli can trigger ltNK cells and more functional experiments are, therefore, required. Altogether, this study confirms the resident nature of ltNK cells and offers opportunities for further studies on the specific function of ltNK cells. Our comprehensive analysis facilitates new strategies to further study tissue-resident features, development of ltNK cells, and their role in immune regulation.

## Data Availability Statement

The dataset generated for this study can be found in the GEO database under accession number GSE116178. To perform comparative analysis, the T cell dataset GSE94964 published by Kumar et al. was used.

## Ethics Statement

This study was carried out in accordance with the recommendations of the Dutch national ethical and profession guidelines. The protocol was approved by the institutional review board. All subjects gave written informed consent in accordance with the Declaration of Helsinki.

## Author Contributions

JM designed the study, performed experiments, analyzed data, and wrote the manuscript. GL designed and performed the RNA sequence experiment and wrote the paper. CV performed flow cytometry experiments. SK analyzed RNA sequence data. SZ analyzed RNA sequence data. HB designed and performed the RNA sequencing. MO contributed expertise and performed flow cytometry experiments. AL and MS supervised the study, designed experiments, and wrote the paper.

## Conflict of Interest Statement

The authors declare that the research was conducted in the absence of any commercial or financial relationships that could be construed as a potential conflict of interest. The reviewer FV and the handling Editor declared their shared affiliation.
