# Supplementary material for: Human Bone Marrow-Resident Natural Killer Cells Have a Unique Transcriptional Profile and Resemble Resident Memory CD8+ T Cells
Source: Front Immunol. 2018 Aug 22;9:1829. doi: 10.3389/fimmu.2018.01829 (PMC6113396; doi:10.3389/fimmu.2018.01829)
Supplement: Supplementary file 1 [file data_sheet_1.PDF]

Supplementary Material

Human Bone Marrow Resident Natural Killer Cells Have a Unique Transcriptional Profile and Resemble Resident Memory CD8$^+$ T Cells

Janine E. Melsen[1*], Gertjan Lugthart[1], Carly Vervat[1], Szymon M. Kielbasa[2], Sander A.J. van der Zeeuw[2], Henk P.J. Buermans[3], Monique M. van Ostaijen-ten Dam[1], Arjan C. Lankester[1], Marco W. Schilham[1]

*** Correspondence:** Janine Melsen: j.e.melsen@lumc.nl

1 Supplementary methods

1.1 Purification of NK cell populations

After mononuclear cell isolation by Ficoll-Isopaque (Leiden University Medical Center, Pharmacy, Leiden, NL), we enriched for NK cells by the MACS untouched NK cell isolation kit ((Miltenyi Biotec, Bergisch Gladbach, DE) according to protocol. NK cell enriched mononuclear cells were stained with unconjugated CXCR6, washed twice and stained with secondary goat anti-mouse IgG2b-Alexa647 in MACS buffer containing 2mM EDTA and 0.5% human serum albumin (HSA, Sanquin, Leiden, NL). Second, cells were stained with directly conjugated antibodies (CD3, CD19, CD56, CD54, CD69, CD16 and NKG2A) in MACS buffer supplied with 5% mouse serum (Seralab, London, UK). All the antibodies used are listed in *Table S1*. Lymphocytes were gated based on forward- and sideward scatter and the doublets were excluded. Residual B- and T cells were excluded by selecting CD19$^-$CD3$^-$ lymphocytes. Lymphoid tissue NK (ltNK) cells were defined as CD56$^+$CXCR6$^+$CD54$^+$CD69$^+$ (Fig. 1A).[21] Conventional NK cells, defined as CD56$^+$CXCR6$^-$, were further divided into CD56^{bright}CD16$^{-/+}$ and CD56^{dim}CD16$^+$. The blood derived CD56^{dim} and bone marrow derived ltNK were further divided into a NKG2A$^+$ and NKG2A$^-$ fraction.

1.2 RNA sequence

The NK cell populations were collected in NucleoSpin RA1 lysis buffer (Machery Nagel, Düren, DE) and stored at -80°C prior to analyses. Total RNA was extracted using the NucleoSpin RNA XS kit (Macherey Nagel), according to the instructions of the manufacturer. RNA concentration was measured using the Qubit (Thermo Fisher Scientific, Waltham, MA, US), and RNA integrity was analyzed using RNA Pico chips on 2100 Bioanalyzer system (Agilent Technologies, Santa Clara, CA, US). The RNA was converted to cDNA and pre-amplified using the SMARTer Ultra Low RNA kit (Clontech Laboratories, Montain View, CA, US) using 0.5-2ng total RNA input. A sequencing library was generated from 1ng (Qubit measured) amplified cDNA using the Illumina Nextera XT kit (Illumina, San Diego, CA, US). Each sample was given a unique sample barcode during this step. All 35 samples were pooled and sequenced on two lanes of the HiSeq2500 system on v4 flowcells and reagents with run type paired-end 2x125bp reads. FastQ files for each sample were generated using CASAVA version 1.8.4 software (Illumina).

1.3 Mapping of reads

In order to detect and remove the sequencing adaptors in the FastQ files, FASTQC version 0.10.0 and the cutadapt tool (version 1.4.2) were used, respectively. Base quality trimming was performed using the sickle tool (version 1.200). RNA paired-end reads were aligned to the *Homo Sapiens* (Human) reference genome version hg19 (GSNAP version 2014-05-15; npaths=1, quit_if_excessive=TRUE and novel splicing has been set to True). Subsequently, the samtools tool (version 0.1.19-44428cd) was applied to compress, index and name-sort the resulting alignment file. The count table, summarizing the total uniquely aligned reads per gene per sample, was generated using htseq-count (HTSeq suite version 0.6.1p1). As a reference, the UCSC genePredToGtf generated RefSeq annotation (raw database dump of the refGene table) was used. This method allows identification of both mRNAs and long non-coding RNAs.

2 Supplementary Figures and Tables

2.1 Supplementary Figures

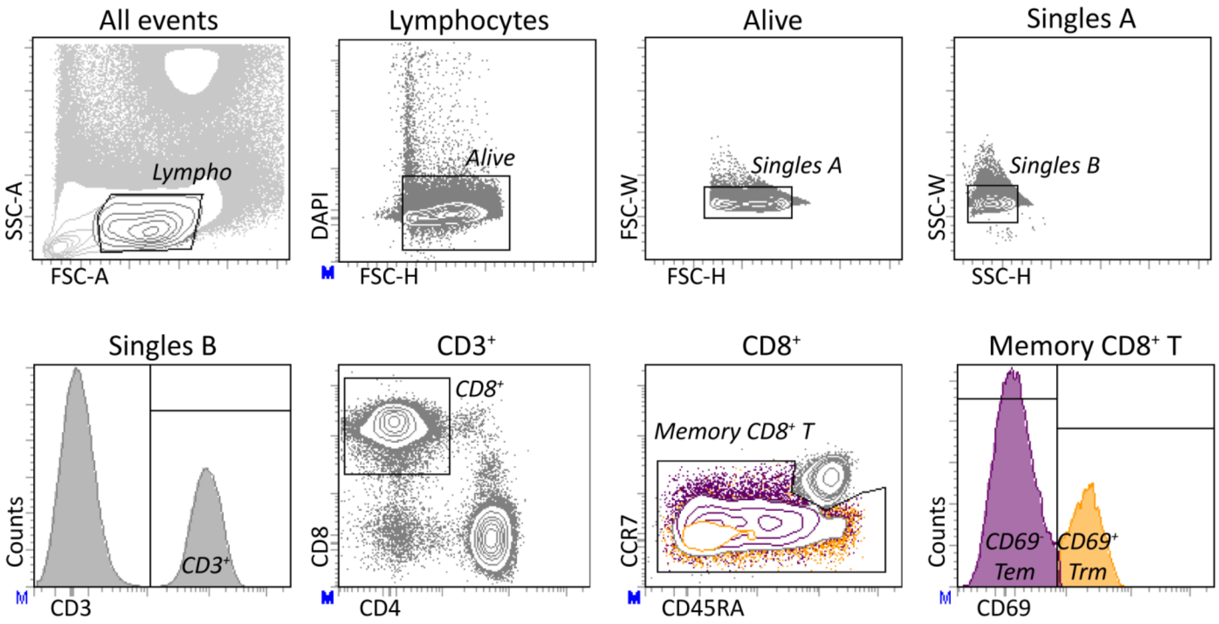

Fig. S1. Gating strategy used to identify tissue-resident CD8$^+$ memory T cells.

First, lymphocytes were selected based on sideward and forward scatter and dead cells were excluded by DAPI. Next, doublets were excluded and CD3$^+$CD8$^+$ T cells were gated. CD8$^+$ memory T cells were identified as CCR7$^-$CD45RA$^{+/-}$. CD69 expression was used to discriminate tissue-resident memory T cells (Trm) from non-resident effector memory T cells (Tem) cells.

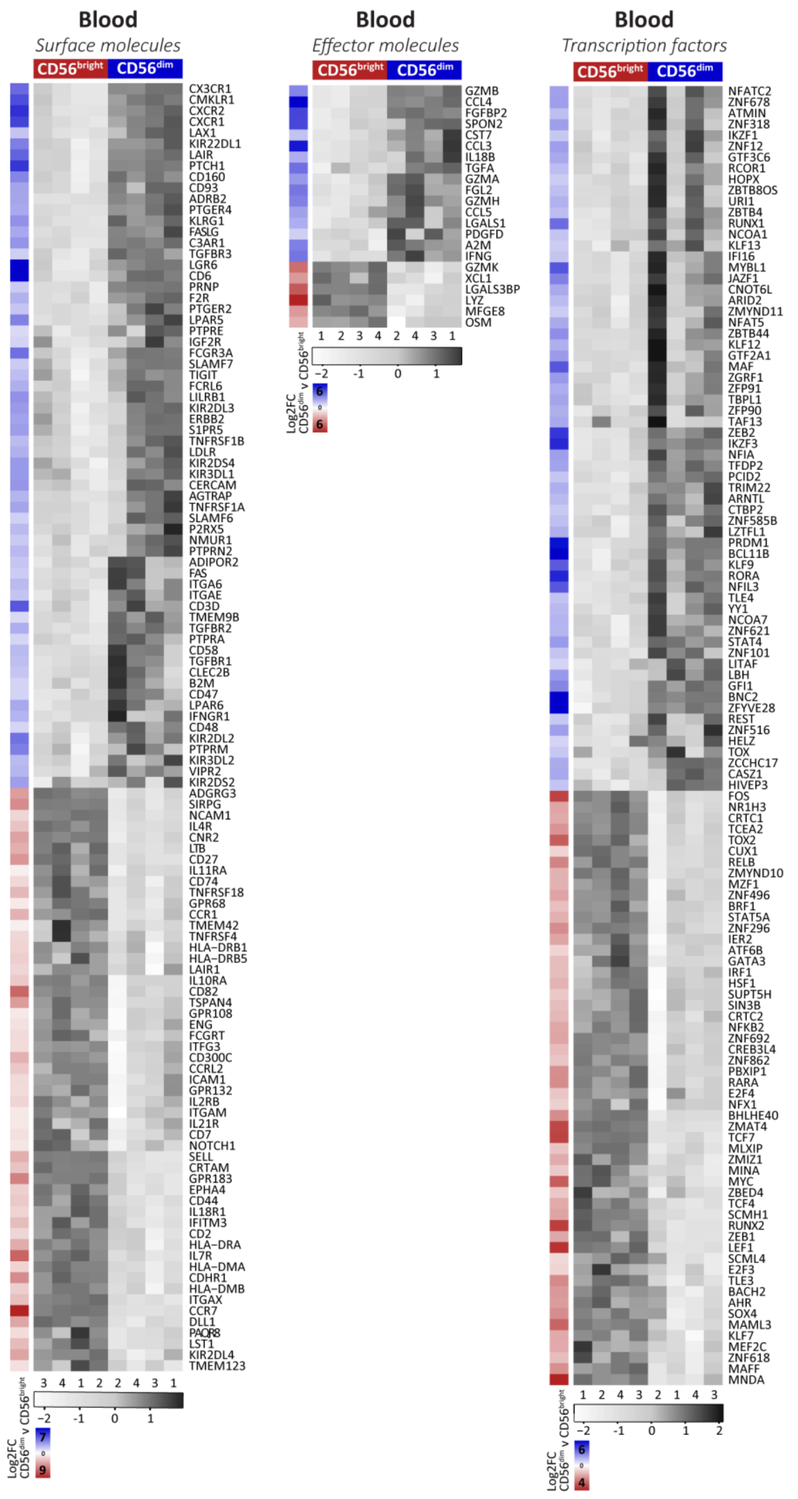

Fig. S2. Heatmaps of genes derived from circulating CD56^{bright} and CD56^{dim} NK cells. Heatmaps show scaled mRNA expression values of genes which encode surface molecules, effector molecules and transcription factors. Genes which have the highest or lowest mRNA expression (false discovery rate<0.05) in blood derived CD56^{bright} or CD56^{dim} NK cells are included. The column side bars represent the log2 fold change (FC) of gene expression levels in CD56^{dim} versus CD56^{bright} NK cells. The color indicates in which NK cell population the gene is expressed at the highest level (red=CD56^{bright}, blue=CD56^{dim}). The color intensity represents the magnitude of the fold change.

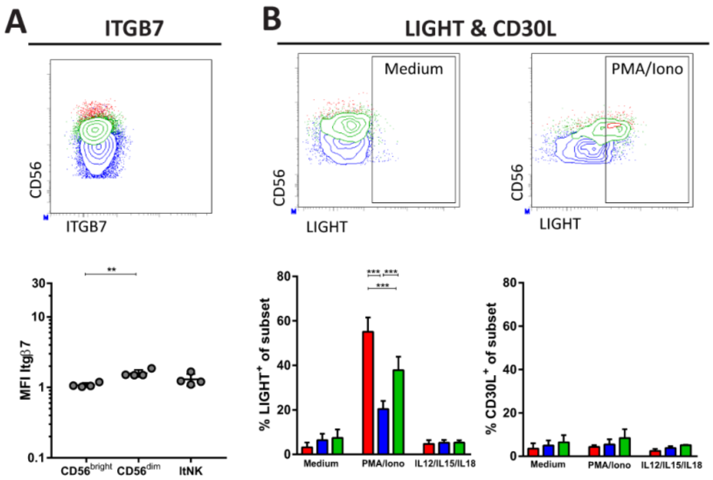

Fig. S3. ITGB7, LIGHT and CD30L expression on NK cell subsets.

(**A**) Protein expression of ITGB7 on bone marrow derived NK cells. **FDR<0.01. FDR = false discovery rate. (**B**) Protein expression of TNFSF14 (LIGHT) and TNFSF8 (CD30L) on cultured bone marrow derived NK cells (n=4) in resting conditions and upon PMA/Ionomycin or IL12/IL15/IL18 stimulation for 4h. Shown are representative dot plots of bone marrow derived NK cells. MFI = mean fluoresence intensity. Mean ± SD are shown. **P<0.01, ***P<0.001, by one-way ANOVA.

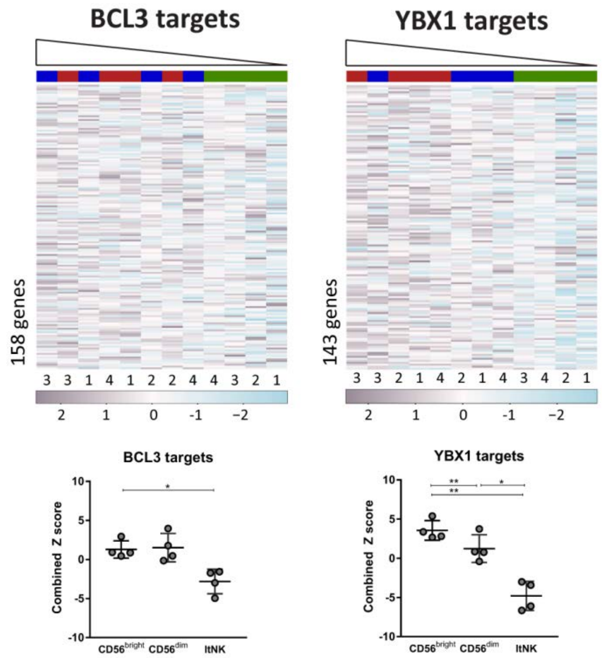

Fig. S4. Gene set enrichment analysis reveals BCL3 and YBX1 as potential regulators of ltNK cell transcriptome.

Gene set enrichment analysis using CAMERA and the Broad institute gene set collections revealed that targets which are repressed by BCL3 (M2424) and YBX1 (M14340) are downregulated in ltNK cells. Heatmaps show the normalized expression values of the corresponding target genes of BCL3 (n=158) and DUSP6 (n=143). The column order is based on the combined Z score of each donor NK cell population (high --> low). The combined Z score is a quantification of the overall expression level of the target genes. Shown are mean ± SD.*P<0.05, **P<0.01, by one-way ANOVA.

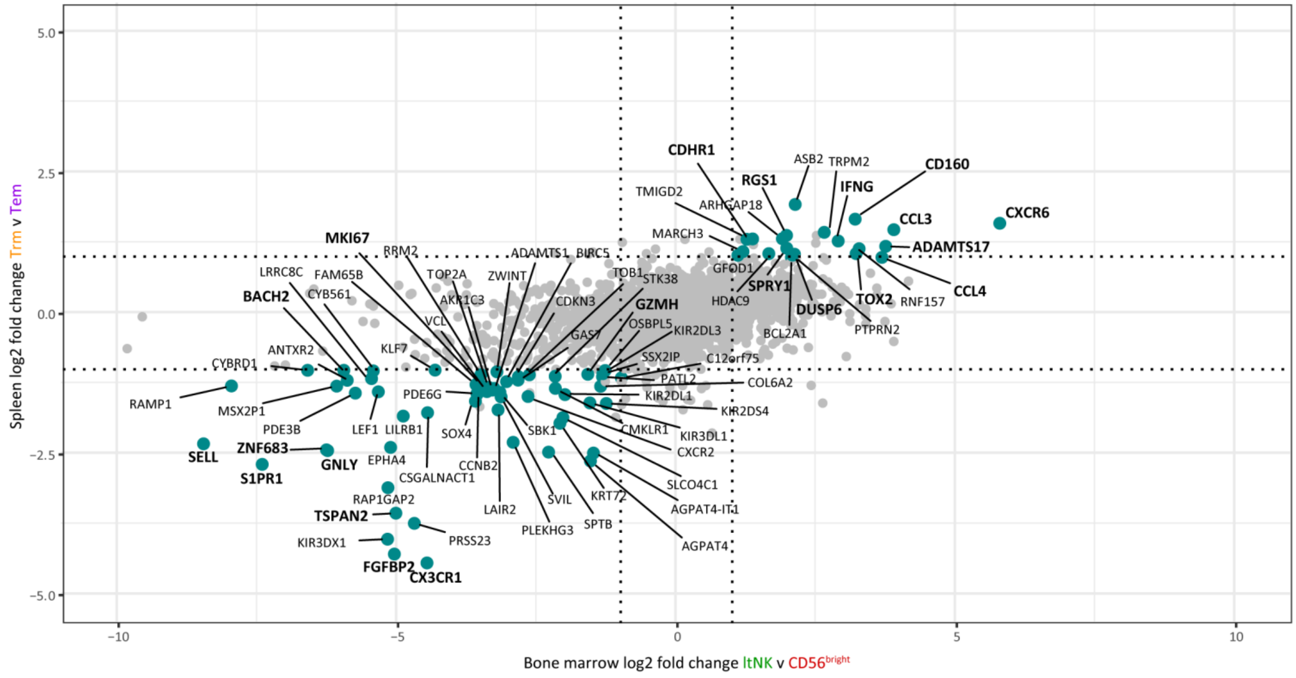

Fig. S5. LtNK cells and tissue-resident $CD8^+$ memory T cells share a core transcriptional profile.

The data set GSE94964 contains RNAseq data of spleen derived effector memory T cells ($CD3^+CD8^+CD45RA^-CCR7^-$) which were subdivided into $CD69^+$ tissue-resident memory (Trm) and $CD69^-$ effector memory T (Tem) cells. We compared this gene set to our RNAseq data to determine a core gene signature among Trm and ltNk cells in lymphoid tissues. The log2 fold change of 8700 genes (which were both expressed in T and NK cells) was calculated. The log2 fold change of ltNK versus $CD56^{bright}$ was plotted against the log2 fold change of $CD8^+$ Trm against Tem cells. Figure 7A depicts the plot which contains the log2 fold change of ltNK versus $CD56^{dim}$. Core resident genes which are depicted in turqoise were identified as genes with a log2 fold change ≥1 or ≤-1 in all three comparisons.

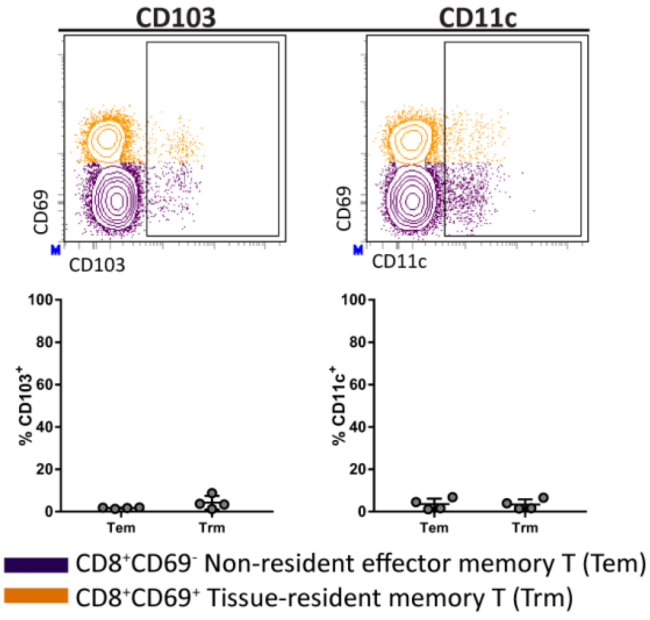

Fig. S6. CD103 and CD11c expression on CD8^{+} memory T cells.

CD103 and CD11c protein expression was determined by flow cytometry on bone marrow derived CD69^{+} and CD69^{-} CD8^{+} memory T cells. Shown is a representative bone marrow donor. Mean ± SD are shown. No significant difference was found, as tested by a paired t-test.

2.2 Supplementary Tables

Table S1. Antibodies used to purify NK cell populations.

Specificity		Antibody characteristics					
CD designation	Alternative name	Fluorochrome	Type	Clone	Company	Catalog#	Dilution
CD186	CXCR6	Unconjugated	m-IgG2b	56811	R&D	MAB699	1:25
Secondary	Goat α m-IgG2b	AF647	polyclonal		Invitrogen	A21242	1:2000
CD3	CD3	BV421	m-IgG1	UCHT1	BD	562426	1:100
CD16	FcγRIII	BV711	m-IgG1	3G8	BD	563127	1:100
CD19	CD19	BV510	m-IgG1	SJ25C1	BD	562947	1:100
CD54	ICAM1	PE	m-IgG1	HA58	BD	555511	1:100
CD56	NCAM1	ECD	m-IgG1	N901	BC	A82943	1:40
CD69	EA-1	FITC	m-IgG1	L78	BD	347823	1:25
CD159a	NKG2A	PE-Cy7	m-IgG2b	z199	BC	PNB10246	1:50

Main Abbreviations: CD: Cluster of differentiation. Fluorochrome: AF: Alexa Fluor, BV: Brilliant Violet, ECD: Energy Coupled Dye (=Phycoerythrin-Texas Red conjugate), FITC: Fluorescein isothiocyanate, PE: Phycoerythrin, PE-Cy7: Phycoerythrin-Cyanine7 conjugate. Type: m: mouse. Company: BC: Beckman Coulter (Brea, CA, USA), BD: Becton Dickinson Biosciences (San Jose, CA, USA), Invitrogen: Invitrogen (Thermo Fisher Scientific, Waltham, MA, USA), R&D: R&D Systems (Minneapolis, MN, USA)

Table S2. Cell numbers of purified NK cell populations.

	Peripheral blood			Bone marrow			
Donor	$CD56^{bright}$	$NKG2A^{-}$ $CD56^{dim}$	$NKG2A^{+}$ $CD56^{dim}$	$NKG2A^{-}$ ltNK	$NKG2A^{+}$ ltNK	$CD56^{bright}$	$CD56^{dim}$
1	14428	10794	15673	16574	54291	12092	402951
2	103198	158296	97570	8348	14331	8282	75731
3	21582	43318	44496	41584	92987	28570	261696
4	68886	165747	132394	123674	157392	56588	411890

Table S3. Antibodies used for RNA sequence validation.

Specificity		Antibody characteristics						
CD designation	Alternative name	Fluorochrome	Type	Clone	Company	Catalog#	Dilution	Cell
CD186	CXCR6	Unconjugated	m-IgG2b	56811	R&D	MAB699	1:25	NK/T
Secondary	Goat α m-IgG2b	AF647	polyclonal		Invitrogen	A21242	1:2000	NK/T
Secondary	Goat α m-IgG2b	PE	polyclonal		Southern	1092-09	1:500	NK/T
CD3	CD3	BV421	m-IgG1	UCHT1	BD	562426	1:200	NK/T
CD3	CD3	BV510	m-IgG1	UCHT1	Biolegend	300448	1:200	NK
CD3	CD3	APC-H7	m-IgG1	SK7	BD	560176	1:25	NK/T
CD4	CD4	PE-Cy5.5	m-IgG1	13B8.2	BC	B16491	1:200	T
CD7	GP40	AF700	m-IgG1	M-T701	BD	561603	1:50	NK
CD7	GP40	APC-R700	m-IgG1	M-T701	BD	659124	1:100	NK
CD8	CD8	BV510	m-IgG1	SK1	BD	563919	1:100	NK/T
CD11c	ITGAX	PE-Cy7	m-IgG1	B-ly6	BD	561356	1:100	NK
CD11c	ITGAX	PE	m-IgG2b	S-HCL-3	BD	333149	1:100	T
CD16	FcγRIII	BV711	m-IgG1	3G8	BD	563127	1:200	NK
CD19	CD19	BV510	m-IgG1	J3.119	BC	IM2470	1:50	NK
CD29	ITGB1	FITC	m-IgG1	4B7R	Bio-Rad	MCA1949F	1:20	NK/T
CD33	Siglec-3	APC	m-IgG1	D3HL60.251	BC	IM24711U	1:150	NK
CD45	PTPRC	PE-Cy5.5	m-IgG1	J33	BC	A54139	1:200	NK
CD45RA	PTPRC	ECD	m-IgG2b	MEM-56	Invitrogen	335039	1:40	T
CD49e	ITGA5	PE	m-IgG1	IIA1	BD	555617	1:50	NK/T
CD54	ICAM1	PE	m-IgG1	HA58	BD	555511	1:100	NK
CD56	NCAM1	BV605	m-IgG1	Leu-19	Biolegend	318334	1:50	NK
CD56	NCAM1	PC5.5	m-IgG1	N901	BC	A79388	1:400	NK
CD56	NCAM1	ECD	m-IgG1	N901	BC	A82943	1:100	NK
CD62L	L-selectin	FITC	m-IgG1	FMC46	DAKO	F7085	1:120	NK/T
CD69	EA-1	FITC	m-IgG1	L78	BD	347823	1:25	NK/T
CD69	EA-1	PE-Cy7	m-IgG1	L78	BD	335792	1:20	NK/T
CD81	TSPAN28	PE	m-IgG1	JS-81	BD	555676	1:20	NK
CD96	Tactile	PE	m-IgG1	6F9	BD	562379	1:20	NK/T
CD103	ITGAE	FITC	m-IgG1	Ber-ACT8	DAKO	F7138	1:20	T
CD186	CXCR6	BV421	m-IgG1	K041E5	Biolegend	356008	1:50	NK
CD186	CXCR6	AF647	m-IgG1	K041E5	Biolegend	356014	1:20	NK
CD197	CCR7	BV711	m-IgG2a	G043H7	Biolegend	353228	1:20	T
CD153/CD30L	TNFSF8	PE	m-IgG2b	116614	R&D	FAB1028P	1:20	NK
CD226	DNAM1	FITC	m-IgG1	DX11	BD	559788	1:5	NK/T
CD258	LIGHT/TNFSF14	AF647	m-IgG1	115520	BD	564374	1:20	NK
	Eomes	eFluor660	m-IgG1	WD1928	eBioscience	50487741	1:20	NK/T
	IFNγ	FITC	m-IgG1	4S.B3	BD	554551	1:25	NK
	Itgβ7	APC	r-IgG2a	FIB504	Biolegend	321207	1:100	NK
	Ki67	FITC	m-IgG1	20Raj1	eBioscience	11569942	1:100	NK
	Tbet	PE	m-IgG1	4B10	Biolegend	644809	1:100	NK/T
	TIGIT	APC	m-IgG1	MBSA43	eBioscience	179500	1:20	NK/T

Main Abbreviations: CD: Cluster of differentiation. Fluorochrome: AF: Alexa Fluor, APC: Allophycocyanin: BV: Brilliant Violet, ECD: Energy Coupled Dye (=Phycoerythrin-Texas Red conjugate), FITC: Fluorescein isothiocyanate, PE: Phycoerythrin, PE-Cy5.5: Phycoerythrin-Cyanine5.5 conjugate, PE-Cy7: Phycoerythrin-Cyanine7 conjugate. Type: m: mouse, r: rat. Company: BC: Beckman Coulter (Brea, CA, USA), BD: Becton Dickinson Biosciences (San Jose, CA, USA), Biolegend: Biolegend (San Diego, CA, USA), Bio-Rad: Bio-Rad laboratories (Hercules, CA, USA), DAKO: Dako Denmark, (Glostrup, Denmark), eBioscience: eBioscience (San Diego, CA, USA),Invitrogen: Invitrogen (Thermo Fisher Scientific, Waltham, MA, USA), R&D: R&D Systems (Minneapolis, MN, USA), Southern: Southern Biotech (Birmingham, AL, USA)

Table S4a. Gene set enrichment analysis using CAMERA: gene sets which are upregulated in ltNK cells versus CD56^{bright} and CD56^{dim} NK cells.

CAMERA analysis was applied by using RNA sequence data and the Broad institue gene collections. Only gene sets which were signifcantly up- or downregulated by ltNK cells versus both CD56^{bright} and CD56^{dim} Nk cells were further analyzed (FDR<0.05). In (**A**) are gene sets included of which the corresponding genes were upregulated by ltNK cells.Downregulated genesets which represent target genes of transcription factors are listed in (**B**). In (**C**) gene sets which include downregulated cell-cycle related genes are listed. Genesets higlighted in grey are visualized in Fig. 5A,B and Sup Fig. S4 .

Geneset	Description	Geneset	N genes/total	ltNK v CD56^{bright} FDR	ltNK v CD56^{dim} FDR
NAGASHIMA_EGF_SIGNALING_UP	Genes up-regulated in MCF7 cells (breast cancer) after stimulation with EGF.	M16311	28/58	2.68E-02	5.46E-05
CROONQUIST_STROMAL_STIMULATION_UP	Genes up-regulated in ANBL-6 cell line (multiple myeloma) co-cultured with bone marrow stromal cells compared to those grown in the presence of IL6.	M5929	16/60	2.13E-03	2.78E-04
AMIT_SERUM_RESPONSE_40_MCF10A	Genes whose expression peaked at 40 min after stimulation of MCF10A cells (breast cancer) with serum.	M11519	16/28	5.96E-03	2.84E-05
BURTON_ADIPOGENESIS_PEAK_AT_2HR	genes maximally expressed at 2 h time point during differentiation of 3T3-L1 fibroblasts into adipocytes in response to adipogenic hormones.	M1597	26/51	1.41E-04	6.54E-05
GO_CYTOKINE_ACTIVITY	Genes encoding cytokines.	M19159	35/219	1.26E-02	1.74E-04
NABA_SECRETED_FACTORS	Genes encoding secreted soluble factors.	M5883	41/344	1.92E-02	2.58E-03
DEBOSSCHER_NFKB_TARGETS_REPRESSED_BY_GLUCOCORTICOIDS	NF-kB-driven pro-inflammatory genes that are negatively regulated by glucocorticoids.	M17340	5/24	4.66E-02	3.19E-03
BIOCARTA_CYTOKINE_PATHWAY	Cytokine network	M17406	6/22	4.35E-02	5.19E-03
GO_CHEMOKINE_ACTIVITY	Genes encoding chemokines.	M18678	6/48	6.32E-04	8.96E-03
GO_LYMPHOCYTE_CHEMOTAXIS	The directed movement of a lymphocyte in response to an external stimulus.	M14280	7/38	3.44E-02	1.12E-02
YE_METASTATIC_LIVER_CANCER	Genes up-regulated in hepatocellular carcinoma with intra-hepatic metastasis compared to the non-metastatic tumors.	M11542	12/27	4.42E-02	1.74E-02
BOQUEST_STEM_CELL_CULTURED_VS_FRESH_DN	Genes downregulated in cultured stromal cells from adiposte tissue versus freshly isolated cells.	M12827	2/30	7.44E-03	2.09E-02
GSE23925_DARK_ZONE_VS_NAIVE_BCELL_DN	Genes down-regulated in B cells: dark zone versus naïve.	M7881	125/200	1.78E-02	2.26E-02
NAKAYAMA_SOFT_TISSUE_TUMORS_PCA1_DN	Top 100 probe sets contributing to the negative side of the 1st principal component; predominantly associated with synovial sarcoma and myxoid/round cell liposarcoma samples.	M17937	6/67	2.24E-02	3.22E-02
GO_RESPONSE_TO_IMMOBILIZATION_STRESS	Any process that results in a change in state or activity of a cell or an organism (in terms of movement, secretion, enzyme production, gene expression, etc.) as a result of being rendered immobile.	M10626	6/22	3.27E-02	3.55E-02
GSE36891_POLYIC_TLR3_VS_PAM_TLR2_STIM_PERITONEAL_MACROPHAGE_UP	Genes up-regulated in peritoneal macrophages: poly(IC) (TLR3 stimulation) versus Pam3Cys-Ser-(Lys)4 (TLR2 stimulation).	M8789	62/146	9.65E-03	4.18E-02
WINZEN_DEGRADED_VIA_KHSRP	Transcripts (mRNA molecules) rapidly degraded upon interaction with KHSRP.	M2352	30/100	1.21E-04	4.67E-02

Table S4b. Gene set enrichment analysis using CAMERA: transcription factor gene sets which are downregulated by ltNK versus CD56^{bright} and CD56^{dim} NK cells.

Transcription factor	Full name	Geneset	N genes/total	Direction	ltNK v CD56^{bright} FDR	ltNK v CD56^{dim} FDR
EGR2 (repressed target genes)	Early Growth Response 2	M12804	73/108	Down	2.79E-16	1.52E-05
BCL3 (repressed target genes)	B-Cell CLL/Lymphoma 3	M2424	158/364	Down	9.94E-03	1.01E-02
YBX1 (repressed target genes)	Y-Box Binding Protein 1	M14340, M14985	143/202, 182/290	Down	2.99E-09, 1.08E-07	1.78E-04, 7.96E-03
NR2E1	Nuclear Receptor Subfamily 2 Group E Member 1	M1916, M1913, M1915	66/89, 146/185, 206/277	Down	1.60E-06, 3.30E-03, 3.30E-03	7.55E-05, 9.63E-04, 1.24E-02
FOXP3	Forkhead Box P3	M1735	55/91	Down	8.80E-13	7.55E-05
E2F1/2/3	E2F Transcription Factor 1/2/3	M1945, M1172, M1157	42/46, 22/31, 60/97	Down	5.94E-20, 2.14E-02, 1.29E-14	2.21E-11, 2.10E-02, 4.79E-07
EZH2	Enhancer Of Zeste 2 Polycomb Repressive Complex 2 Subunit	M1486	38/41	Down	5.00E-10	5.66E-06
TP53	Tumor Protein P53	M6171, M9402	36/57, 19/22	Down	2.35E-10, 1.08E-09	9.24E-06, 3.20E-04
MYC	C-myc	M1249, M6792	34/42, 30/45	Down	2.35E-10, 4.88E-07	9.96E-05, 1.23E-02
MYBL2	MYB Proto-Oncogene Like 2	M15973, M11840	31/74, 85/200	Down	3.59E-08, 1.30E-06	4.37E-05, 4.75E-03
FOXO3	Forkhead Box O3	M2314	30/41	Down	6.31E-09	2.05E-03
WTAP	Wilms Tumor 1 Associated Protein	M10279	215/310	Down	1.81E-06	4.17E-03
TCF3	Transcription factor 3	M1490	21/33	Down	1.18E-10	4.87E-04
LIN9	Lin-9 DREAM MuvB Core Complex Component	M2483	18/28	Down	6.84E-07	2.74E-04
ETV5	ETS Variant 5	M1664	14/23	Down	4.36E-03	1.09E-02
RB1	Retinoblastoma 1	M2128, M4455, M2129	121/243, 17/23, 241/567	Down	1.52E-07, 3.84E-09, 7.27E-04	1.69E-03, 2.24E-03, 1.01E-02
PROX1	Prospero Homeobox 1	M1495	11/28	Down	2.40E-05	4.28E-06
YY1	Yin and Yang 1 Transcription Factor	M1471	104/234	Down	6.55E-05	4.92E-04

Table S4c. Gene set enrichment analysis using CAMERA: cell-cyle related gene sets which are downregulated in ltNK cells versus CD56^{bright} and CD56^{dim} NK cells.

Geneset	Description	Geneset	N genes/total	ltNK v CD56bright FDR	ltNK v CD56dim FDR
HALLMARK_E2F_TARGETS	Genes encoding cell cycle related targets of E2F transcription factors.	M5925	164/200	3.59E-09	2.08E-04
HALLMARK_G2M_CHECKPOINT	Genes involved in the G2/M checkpoint, as in progression through the cell division cycle.	M5901	158/200	2.66E-06	1.33E-04
BENPORATH_PROLIFERATION	Set 'Proliferation Cluster': genes defined in human breast tumor expression data.	M2114	118/147	1.19E-05	2.92E-05
CHANG_CYCLING_GENES	Fibroblast serum response genes showing periodic expression during the cell cycle; excluded from the core serum response signature.	M11537	96/148	1.61E-09	6.54E-05
EGUCHI_CELL_CYCLE_RB1_TARGETS	RB1 target genes involved in cell cycle regulation: genes down-regulated by doxorubicin only in cells expressing RB1.	M4455	17/23	3.84E-09	2.24E-03
GRAHAM_NORMAL_QUIESCENT_VS_NORMAL_DIVIDING_D	Genes down-regulated in quiescent vs dividing CD34+ cells isolated from peripheral blood of normal donors.	M5198	53/83	3.33E-20	3.41E-08
REACTOME_CELL_CYCLE	Genes involved in Cell Cycle.	M543	303/421	1.91E-02	2.99E-02
REACTOME_CELL_CYCLE_MITOTIC	Genes involved in Cell Cycle, Mitotic.	M5336	257/325	6.22E-03	2.73E-02
REACTOME_DNA_REPLICATION	Genes involved in DNA Replication.	M1017	158/192	1.18E-03	4.86E-03
REACTOME_G1_S_SPECIFIC_TRANSCRIPTION	Genes involved in G1/S-Specific Transcription.	M1040	6/19	6.53E-05	5.99E-04
REACTOME_G1_S_TRANSITION	Genes involved in G1/S Transition.	M17283	87/112	2.49E-02	3.14E-02
REACTOME_MITOTIC_M_M_G1_PHASES	Genes involved in Mitotic M-M/G1 phases.	M7634	143/172	1.38E-03	2.71E-03
REACTOME_MITOTIC_PROMETAPHASE	Genes involved in Mitotic Prometaphase.	M4217	73/87	4.36E-03	8.41E-03
REACTOME_REGULATION_OF_MITOTIC_CELL_CYCLE	Genes involved in Regulation of mitotic cell cycle.	M851	74/85	9.57E-03	7.41E-03
REICHERT_MITOSIS_LIN9_TARGETS	Genes with known mitosis function that were down-regulated in MEF cells (embryonic fibroblast) upon knockout of LIN9.	M2483	18/28	6.84E-07	2.74E-04
SCIAN_CELL_CYCLE_TARGETS_OF_TP53_AND_TP73_DN	Cell cycle genes down-regulated in H1299 cells (lung cancer) after overexpression of either P53 or P73.	M9402	19/22	1.08E-09	3.20E-04
WHITFIELD_CELL_CYCLE_LITERATURE	A list of known cell cycle regulated genes that was compiled from the literature by the authors.	M2066	28/44	1.16E-15	1.37E-07
ZHOU_CELL_CYCLE_GENES_IN_IR_RESPONSE_24HR	Cell cycle genes significantly (p =< 0.05) changed in fibroblast cells at 24 h after exposure to ionizing radiation.	M2608	58/128	6.18E-10	7.05E-05
ZHOU_CELL_CYCLE_GENES_IN_IR_RESPONSE_6HR	Cell cycle genes significantly (p =< 0.05) changed in fibroblast cells at 6 h after exposure to ionizing radiation.	M2606	56/85	1.20E-07	2.67E-04
EGUCHI_CELL_CYCLE_RB1_TARGETS	RB1 target genes involved in cell cycle regulation: genes down-regulated by doxorubicin only in cells expressing RB1.	M4455	17/23	3.84E-09	2.24E-03

Table S5. Core gene signature among tissue-resident CD8+ memory T cells in spleen and ltNK cells in bone marrow.
Genes with a log2 fold change ≤-1 or ≥ 1 in CD8 Trm v Tem, ltNK v $CD56^{dim}$ and ltNK v $CD56^{bright}$ were selected.

Genename	Direction	Spleen CD8 Trm v Tem	Bone marrow ltNk v $CD56^{dim}$	Bone marrow ltNK v $CD56^{bright}$
**ADAMTS1**	Down	-1.32668179137151	-3.52425497016591	-3.28104038944956
**ADAMTS17**	Up	1.19989749199077	6.67398463080119	3.72755501942304
**AGPAT4**	Down	-2.61435596255051	-1.70565655093335	-1.55908807808568
**AGPAT4-IT1**	Down	-2.47474126812555	-1.89436308037301	-1.50476078772464
**AKR1C3**	Down	-1.35586163650925	-8.49315300096715	-3.31681837016608
**ANTXR2**	Down	-1.18033979059349	-4.48357324949886	-5.90925972632214
**ARHGAP18**	Up	1.33594888283704	1.39879868728823	1.87694358088937
**ASB2**	Up	1.94394581965877	1.75289795639305	2.10705370636382
**BACH2**	Down	-1.00344841750256	-4.48471388151011	-5.96973907249895
**BCL2A1**	Up	1.00337741585554	1.46862755933843	2.04142298576875
**BIRC5**	Down	-1.20320171439633	-2.30694190345772	-3.05886097910168
**C12orf75**	Down	-1.13967008374047	-2.63311108778919	-1.00204866114622
**CCL3**	Up	1.49645034349050	4.40534757733708	3.87280277129793
**CCL4**	Up	1.00390837726934	1.62829506729373	3.65888759737754
**CCNB2**	Down	-1.42999626414437	-2.32743285696506	-3.58466809325546
**CD160**	Up	1.68313070271883	1.88565427252163	3.18079197324477
**CDHR1**	Up	1.33225110388469	6.12319927746246	1.34258548718225
**CDKN3**	Down	-1.11803679159409	-2.53329321630581	-2.84711807684210
**CMKLR1**	Down	-1.32629836776899	-9.34557537513476	-2.18569798657033
**COL6A2**	Down	-1.28923236481084	-3.73668942735849	-1.37543180763677
**CSGALNACT1**	Down	-1.75703591484935	-2.62652673766590	-4.47345176318997
**CX3CR1**	Down	-4.42659314417275	-9.31923289130135	-4.48426427701489
**CXCR2**	Down	-1.46855370935076	-6.54319595012704	-2.67539013114242
**CXCR6**	Up	1.61021758213614	7.82237316910911	5.76887111551487
**CYB561**	Down	-1.01493864327682	-3.61098321325399	-5.44316083558976
**CYBRD1**	Down	-1.00168555896364	-7.52827318804288	-6.61792841156806
**DUSP6**	Up	1.05826249178674	2.01528408209472	2.09459621943614
**EPHA4**	Down	-2.37466056200599	-3.89966934553901	-5.13486803615574
**FAM65B**	Down	-1.25987830312636	-3.53731713150736	-3.60591808841292
**FGFBP2**	Down	-4.27014236471812	-9.97244709030317	-5.06852711821301
**GAS7**	Down	-1.17613941081039	-2.15997853753652	-2.85442570835785
**GFOD1**	Up	1.03905489238802	1.21287147268291	1.08164802884837
**GNLY**	Down	-2.42876702993540	-6.21911665909145	-6.26078019171125
**GZMH**	Down	-1.07362243565533	-5.03378841502254	-1.60492434237006
**HDAC9**	Up	1.07127956584090	2.86598628539916	1.63473640818046
**IFNG**	Up	1.29501856975873	1.77563735808190	2.87625358034960
**KIR2DL1**	Down	-1.43608699721888	-5.69700198724012	-2.01319328444659
**KIR2DL3**	Down	-1.00667516610544	-4.35620195378497	-1.29422799076634
**KIR2DS4**	Down	-1.59329807322312	-4.47163648173321	-1.27272631087577
**KIR3DL1**	Down	-1.58311164427484	-4.19688859261553	-1.56762851481901
**KIR3DX1**	Down	-4.00356020578506	-6.09088636533916	-5.18943622717933
**KLF7**	Down	-1.00138543065872	-3.23422746240392	-4.33705781818078
**KRT72**	Down	-1.94386733790432	-6.34157895437522	-2.10380440787255
**LAIR2**	Down	-1.70520883688855	-8.22552589713776	-3.21277207273721
**LEF1**	Down	-1.38392952072986	-1.74342429984811	-5.35479779099345
**LILRB1**	Down	-1.81811413608369	-6.09442362532280	-4.90906528476979
**LRRC8C**	Down	-1.15080681620090	-5.32300306668598	-5.47512812194805
**MARCH3**	Up	1.10617760683086	1.75099053342786	1.17336245044421
**MKI67**	Down	-1.28375839569495	-3.21613806459069	-3.50047869342989
**MSX2P1**	Down	-1.28579967512616	-6.08543623390050	-6.09967615239497
**OSBPL5**	Down	-1.00578025279627	-1.65087711420781	-1.29507551333928
**PATL2**	Down	-1.11950827539058	-2.20889096859470	-1.34291419466163
**PDE3B**	Down	-1.40855617748192	-6.46321209509078	-5.76521956978947
**PDE6G**	Down	-1.41701172494193	-2.14264367424123	-3.58940285090035

**PLEKHG3**	Down	-2.28238034531064	-3.66863905337898	-2.94167836859941
**PRSS23**	Down	-3.72253639094837	-8.13950813421700	-4.70844577638763
**PTPRN2**	Up	1.04371990031832	1.07847596986466	2.06206163575412
**RAMP1**	Down	-1.28283015457869	-7.32823593358245	-7.98399134562260
**RAP1GAP2**	Down	-3.08998777189007	-6.56416378960421	-5.18291774199509
**RGS1**	Up	1.39651868495674	2.97468347069714	1.95336800565128
**RNF157**	Up	1.15761888194982	1.30191353887614	3.25222488212820
**RRM2**	Down	-1.08038164576273	-4.04602353518983	-3.51867062166963
**S1PR1**	Down	-2.67484659567144	-6.98153770936766	-7.43300496909394
**SBK1**	Down	-1.39603555598813	-5.13237567763674	-3.17635360524217
**SELL**	Down	-2.30976737130859	-5.38894825003166	-8.48370106959096
**SLCO4C1**	Down	-1.84413340201013	-4.03425688656136	-2.05232805723711
**SOX4**	Down	-1.55312389421216	-1.01886003407452	-3.61769775631403
**SPRY1**	Up	1.16528128979266	5.71157177397722	1.95538051060526
**SPTB**	Down	-2.45705397375220	-3.26597086718896	-2.30695749973193
**SSX2IP**	Down	-1.06467084619554	-2.48371532269264	-1.33275135222658
**STK38**	Down	-1.10879784739494	-2.92511687712700	-2.18770485086066
**SVIL**	Down	-1.47631727954082	-2.84769868410155	-3.15699844376942
**TMIGD2**	Up	1.32516494125966	1.40096952619095	1.24561372654353
**TOB1**	Down	-1.08580430701613	-1.54823167006575	-2.65039835271743
**TOP2A**	Down	-1.30645205871255	-2.86493044013273	-3.38414917870849
**TOX2**	Up	1.07050118501898	6.35621584740901	3.19704420628145
**TRPM2**	Up	1.44767866553315	2.47022003433563	2.62487421139104
**TSPAN2**	Down	-3.54449571036316	-4.52476116790489	-5.04171174053687
**VCL**	Down	-1.38291282222146	-4.48761345792180	-3.41045069076593
**ZNF683**	Down	-2.41586449402602	-6.72904429984608	-6.28066833593188
**ZWINT**	Down	-1.03148018678417	-2.23742478869097	-3.23107397611972
